# *N*‑Hydroxyphthalimide Dissolved in Ionic Liquids Supported on Carbon Nanotubes as a Hybrid Catalytic System for Solvent-Free Aerobic Oxidation of Ethylbenzene

**DOI:** 10.1021/acsomega.5c01715

**Published:** 2025-10-07

**Authors:** Shakir Ul Azam, Beata Orlińska, Kamil Peckh

**Affiliations:** Department of Chemical Organic Technology and Petrochemistry, 49569 Silesian University of Technology, 44-100 Gliwice, Poland

## Abstract

Carbon nanotubes have gained significant interest in catalysis (as catalysts and catalyst supports) for hydrocarbon oxidation processes. In this study, pristine multiwalled carbon nanotubes and copper­(II) functionalized multiwalled carbon nanotubes were coated with [bmim] cationic ionic liquids (ILs) containing dissolved *N*-hydroxyphthalimide (NHPI) to produce novel SILP and SCILL-SILP hybrid catalytic systems, respectively (SILP: supported ionic liquid phase and SCILL: solid catalyst with an ionic liquid layer). The catalytic activities of the produced systems were investigated for the solvent-free oxidation of ethylbenzene (EB) (80 °C, 0.1 MPa, 6 h) using molecular oxygen as a green oxidant. Among the SILP systems, the 1-butyl-3-methylimidazolium chloride ([bmim]­[Cl])-based SILP system exhibited the highest conversion of EB (12.2 ± 3.1%) with enhanced selectivity (84.1 ± 11.4%) toward acetophenone (AcPO). The catalytic activity of the SILP system increased with increasing lipophilicity of the alkyl group in the IL cation. Conversely, among the SCILL-SILP systems, the highest conversion of EB (22.6 ± 1.2%) was achieved using 1-butyl-3-methylimidazolium bis­(trifluoromethylsulfonyl)­imide ([bmim]­[NTf_2_]) as the IL phase. Recyclability and reusability studies showed that the catalytic activities of the SILP and SCILL-SILP hybrid systems generally decreased in the subsequent cycles, except for 1-butyl-3-methylimidazolium octyl sulfate ([bmim]­[OcOSO_3_])-based catalytic systems, which were relatively stable.

## Graphical abstract

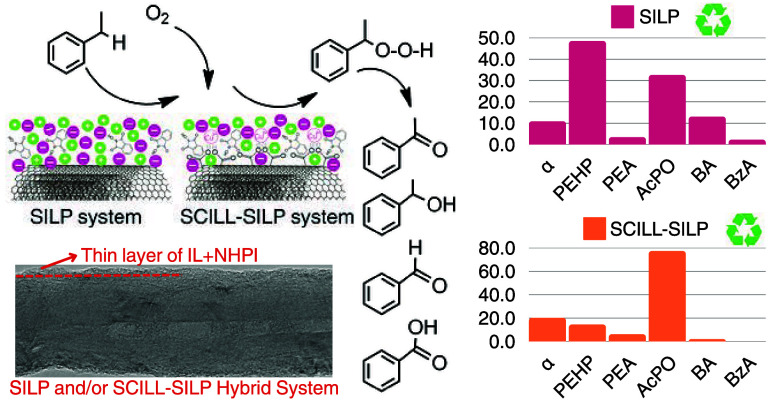

## 1 Introduction

Carbon materials, particularly carbon nanotubes (CNTs), have gained significant interest in various fields, including catalysis, due to their unprecedented physical and chemical properties, such as low cost, environmental friendliness, elevated conductivity, high specific surface area, and tunable chemical functionality.^1,2^ Among various catalytic processes, the oxidation of hydrocarbons to more valuable oxygenated compounds, such as hydroperoxides, alcohols, ketones, aldehydes, acids, and epoxides, is of crucial importance.^3,4^ Transition-metal salts and complexes have been employed as catalysts in these processes;^5,6^ however, the required conditions are harsh, and the processes have limited selectivity. In addition, the separation of homogeneous catalysts from the reaction mixture is challenging, leading to product contamination. Furthermore, toxic metal oxides,^7^ peroxides,^8–10^ and ozone^11^ have been utilized as oxidants in these processes; however, molecular oxygen is known as a greener alternative.^12^ Organic solvents have also been employed frequently to enhance the efficiency of these processes, but their use raises environmental and economic concerns because of issues related to solvent toxicity, flammability, and recovery.^13^ Consequently, the implementation of a more sustainable and environmentally friendly alternative catalytic process becomes imperative. Carbon nanotubes could serve as heterogeneous catalysts and catalytic supports in these oxidation processes as a greener alternative, with molecular oxygen as an oxidant.

Even though silica and alumina have been widely explored as catalyst supports in hydrocarbon oxidation reactions,^14–18^ they are electrically insulating^19–21^ and do not participate in electron transfer processes. In contrast, CNTs have been demonstrated not only as efficient catalyst supports but also as active metal-free catalysts in hydrocarbon oxidation processes, owing to their remarkable electrical conductivity, which promotes efficient electron transfer between active sites and reactants.^22^ Additionally, research has revealed that the superior catalytic activities of sp^2^ carbons, such as graphene and CNTs, are attributed to their ability to accelerate the decomposition of intermediate hydroperoxides in the liquid-phase aerobic oxidation of hydrocarbons.^23^ The transfer of electrons from the graphene sheets of CNTs is a crucial factor in determining their catalytic activity in free-radical chain reactions, as observed in the case of hydrocarbon oxidation in the liquid phase. The delocalized electrons on the graphene sheets of pristine CNTs facilitate π–π interactions between radicals and intermediate hydroperoxides, leading to the decomposition of intermediate hydroperoxides into free radicals, thus improving the overall catalytic activity.^24^ For example, Luo et al.^25^ demonstrated the catalytic activity of pristine CNTs as metal-free catalysts in the aerobic oxidation of ethylbenzene (EB) to acetophenone (AcPO). The enhanced catalytic activity was attributed to the capability of CNTs to expedite the decomposition of intermediate hydroperoxides, a result of the π–π interactions between the delocalized electrons on the CNTs surface and the intermediate hydroperoxide, as well as the free radicals. The same phenomenon was observed in the case of cumene oxidation to cumene hydroperoxide using CNTs as a catalyst.^26^

Functionalization of CNTs is another advantageous way to tailor their catalytic properties.^27,28^ Functionalized CNTs have been extensively investigated for the liquid-phase aerobic oxidation of hydrocarbons. Yu et al.^24^ studied the catalytic activities of multiwalled CNTs (MWCNTs) and nitrogen-doped CNTs (N-CNTs) in the aerobic oxidation of hydrocarbons in the liquid phase. The N-CNTs displayed remarkable performance compared to pristine MWCNTs due to nitrogen doping, which facilitated electron transfer in the graphene sheet. Cao et al.^29^ also investigated the catalytic activities of heteroatom-doped CNTs using nitrogen (N), phosphorus (P), and boron (B) as heteroatoms. N-CNTs and P-CNTs performed quite well, whereas B-CNTs were not active in the liquid-phase aerobic oxidation of cyclohexane. Researchers have found that doping heteroatoms, for example, nitrogen doping in CNTs, elevates the interactions between radicals, intermediate hydroperoxides, and the graphene sheets of CNTs, which in turn augments the catalytic activity of the modified CNTs.^30–33^ In addition, heteroatom doping accelerates the decomposition of intermediate hydroperoxides, which again improves their catalytic activity.^34–36^

The catalytic activity of CNTs in the liquid-phase aerobic oxidation of hydrocarbons can be further enhanced by improving the surface electron transfer of CNTs through metal functionalization. This involved the incorporation of metals such as Co, Fe, Pd, Au, etc.^37–40^ with CNTs. Various metallic compounds, including metallic salts of Cu­(II), Co­(II), and Mn­(II),^41^ metal oxides such as MnO_2_ ^42^ and Mn_3_O_4_,^43^ metal complexes such as Pt and Pd pincer complexes,^44^ and Mn­(III) porphyrins,^45^ among others, have also been used in the functionalization of CNTs to augment their catalytic activity in the liquid-phase aerobic oxidation of hydrocarbons. Additionally, metal nanoparticles and/or nanocatalysts have been confined in CNTs.^46,47^ The resultant metal-confined and/or filled CNTs have displayed improved catalytic activities in the oxidation of hydrocarbons.^38,48–51^ Moreover, CNTs and functionalized CNTs were stable and recyclable in all of the above-reported cases. In addition, researchers have reported the improved catalytic activity of *N*-hydroxyphthalimide (NHPI) in hydrocarbon oxidation reactions.^52–56^ In fact, the superior catalytic activity of NHPI is associated with the formation of the phthalimide *N*-oxyl radical (PINO) (Scheme 1), which can abstract hydrogen atoms from hydrocarbons more effectively than peroxyl radicals in autocatalytic processes.^17^ A variety of additives, such as azo compounds, peroxides, transition metal salts, aldehydes, quinones, and their derivatives, are used to generate the PINO radical.^57^

**Scheme 1:**
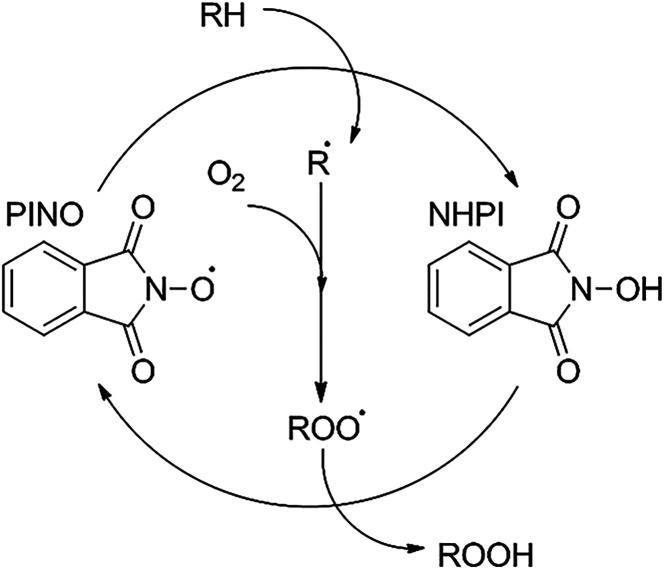
Reaction Mechanism of Hydrocarbon Oxidation in the Presence of NHPI^a^

a Redrawn from ref 58.

Peckh et al.^41^ studied the impact of NHPI as a cocatalyst in the presence of azobisisobutyronitrile (AIBN) (0.2 mol %) as a radical initiator with CNTs functionalized by transition metal salts of Cu­(II), Co­(II), and Mn­(II) for the liquid phase aerobic oxidation of EB (80 °C, 0.1 MPa, 6 h). Following the introduction of 0.5 mol % NHPI, the catalytic activity increased by 2.3, 3.4, and 1.6 times for MWCNT-COO-Co, MWCNT-COO-Cu, and MWCNT-COO-Mn, respectively. The redox potential of the transition metal salts follows the order: Co­(III)/Co­(II) (1.82 V) > Mn­(III)/Mn­(II) (1.51 V) > Cu­(II)/Cu­(I) (0.16 V). Nonetheless, the Cu­(II)-functionalized CNTs exhibited higher catalytic activity, which could be attributed to their higher capability to decompose the intermediate hydroperoxides into the corresponding ketones at a higher rate compared to Co­(II) and Mn­(II) functionalized CNTs.^59,60^ It is also evident from the literature that the use of ionic liquids (ILs) can improve the catalytic performance of NHPI.^60–65^ For example, when [bmim]­[OcOSO_3_] was added to the NHPI/Co­(II) system during the aerobic oxidation of EB (80 °C, 6 h, 0.1 MPa), the conversion increased from 14.9% to 24.5%.^64^ The idea of a “supported ionic liquid phase (SILP)”, in which a thin layer of IL is coated over a solid support, was proposed by Mehnert et al.^66,67^ and Riisager et al.^68,69^ to heterogenize ILs. As a “solid catalyst with ionic liquid layer (SCILL)”, ILs have also been reported to be coated over solid catalysts.^70,71^ Both the SILP and SCILL systems combine the benefits of homogeneous catalysts, such as enhanced catalytic activity, and heterogeneous catalysts, such as ease of catalyst separation and recycling.

Dobras et al.^72^ investigated the catalytic activity of various SCILL systems for the solvent-free oxidation of EB using molecular oxygen as an oxidant (80 °C, 0.1 MPa, 6 h). SCILL systems were prepared by coating different ionic liquids (ILs), such as [bmim]­[OcOSO_3_], [bmim]­[Cl], and [bmim]­[CF_3_SO_3_], containing dissolved CoCl_2_ onto NHPI-immobilized silica gel. The results revealed that all SCILL systems converted EB more efficiently than the system comprising only a mixture of IL and CoCl_2_. The highest conversion of EB (12.1%) was obtained for the [bmim]­[OcOSO_3_]-based SCILL system. The recyclability data showed that the [bmim] cation-based SCILL system, containing [OcOSO_3_] and [CF_3_SO_3_] anions showed a drop in conversion after the fourth cycle, whereas the conversion of the [bmim]­[Cl]-based SCILL system dropped after the third cycle, which was due to the leaching of the mixture of IL and CoCl_2_ from the solid support surface. Talik et al.^73^ coated a mixture of IL, [emim]­[OcOSO_3_], and CoCl_2_ onto an NHPI-immobilized polystyrene support. The resultant SCILL/SILP catalytic system was then employed for the oxidation of EB using dioxygen as an oxidant in a solvent-free environment (80 °C, 0.1 MPa, 6 h). The SCILL/SILP (PS-NHPI-4@CoCl_2_@[emim]­[OcOSO_3_]) system achieved a 7.2% conversion of EB with significantly improved selectivity (76.5%) toward AcPO. The recyclability data indicated that the SCILL/SILP system could be reused without a significant loss in catalytic performance.

In our previous research,^74^ we developed a SCILL and SCILL-SILP hybrid catalytic system utilizing [emim]­[OcOSO_3_] IL, which was chosen for its remarkable catalytic performance and stability, as demonstrated by repeated recyclability in the SCILL/SILP system developed by Talik et al.^73^ The SCILL system was prepared by coating IL, [emim]­[OcOSO_3_], onto Cu­(II) immobilized industrial-grade multiwalled carbon nanotubes (MWCNT-COO-Cu), while the SCILL-SILP hybrid catalytic system was prepared by coating a thin layer of dissolved NHPI in IL ([emim]­[OcOSO_3_]) onto MWCNT-COO-Cu. The synthesized catalytic systems were then applied for the solvent-free oxidation of EB using molecular oxygen as an oxidant (80 °C, 0.1 MPa, 6 h). It was observed that coating only IL over the MWCNT-COO-Cu, as in the case of the SCILL system, reduced the catalytic activity due to the high viscosity of IL, which hindered the radical formation required to initiate the oxidation reaction. Compared with previous studies, the SCILL-SILP hybrid catalytic system exhibited impressive catalytic activity with 27% EB conversion and 77% selectivity toward AcPO. However, the recyclability test of the SCILL-SILP system revealed that part of the mixture of NHPI and IL was leached out, which reduced its catalytic performance in the corresponding cycles.

In this study, an investigation has been conducted to produce more resilient and recyclable SILP and SCILL-SILP hybrid catalytic systems with the synergetic effects of CNTs and/or functionalized CNTs, ILs, and NHPI for the solvent-free aerobic oxidation of EB using molecular oxygen as a green oxidant. The SILP systems were prepared by coating NHPI dissolved in ILs over pristine MWCNTs, whereas the SCILL-SILP hybrid catalytic systems were prepared by coating NHPI dissolved in ILs over MWCNT-COO-Cu. To the best of our knowledge, the reported SILP and SCILL-SILP hybrid catalytic systems are novel and have not been reported previously except for the [emim]­[OcOSO_3_]-based SCILL-SILP system, which we reproduced just for the sake of comparison from our previous work. The recyclability and reusability of the SILP and SCILL-SILP catalytic systems have also been thoroughly examined.

## 2 Experimental Section

### 2.1 Materials

Ethylbenzene (EB) (99.8%), purchased from Acros Organics, was washed with sulfuric acid and distilled under a vacuum. Multiwalled carbon nanotubes (MWCNTs) with purity = 95+ wt%, outer diameter (OD) = 20–40 nm, and length (*L*) = 1–2 μm, and carboxylated multiwalled carbon nanotubes (MWCNT-COOH) with purity = 95+ wt%, OD = 8–15 nm, and *L* = 10–50 μm were purchased from IOLITEC Ionic Liquids Technologies GmbH, Heilbronn, Germany. Copper chloride dihydrate (CuCl_2_·2H_2_O) was purchased from POCH, Poland. Ammonia solution (25%), acetone (99%), and ethanol (99.8%) were purchased from Chempur, Poland. Acetone was dried over molecular sieves. *N*-hydroxyphthalimide (NHPI) and nylon membrane filters (pore size 0.22 μm and diameter 47 mm) were purchased from Sigma-Aldrich, Steinheim, DE, USA. Ionic Liquids: 1-butyl-3-methylimidazolium chloride ([bmim]­[Cl]), 1-butyl-3-methylimidazolium bromide ([bmim]­[Br]), 1-butyl-3-methylimidazolium octylsulfate ([bmim]­[OcOSO_3_]), 1-butyl-3-methylimidazolium hexafluorophosphate ([bmim]­[PF_6_]), 1-butyl-3-methylimidazolium tetrafluoroborate ([bmim]­[BF_4_]), 1-butyl-3-methylimidazolium bis­(trifluoromethylsulfonyl)­imide ([bmim]­[NTf_2_]), 1-ethyl-3-methylimidazolium bis­(trifluoromethanesulfonyl)­imide ([emim]­[NTf_2_]), 1-ethyl-3-methylimidazolium octylsulfate [emim]­[OcOSO_3_], 1-octyl-3-methylimidazolium chloride ([omim]­[Cl]), and 1-decyl-3-methylimidazolium chloride ([C_10_mim]­[Cl]), were commercial materials and were dried under vacuum before use (50 °C, 0.01 MPa).

### 2.2 Preparation of MWCNT-COO-Cu

MWCNT-COO-Cu was synthesized according to a previously defined technique.^41,74^ Initially, 1 g of MWCNT-COOH was added to 40 mL of a 10% aqueous ammonia solution. The mixture was then sonicated for 3 h, filtered, thoroughly washed with deionized water, and dried at 90 °C for 3 h to produce ∼1 g of MWCNT-COO-NH_4_. 0.2 g of the obtained MWCNT-COO-NH_4_ was combined with 25 mL of ethanol, to which 5 mL of a 0.3 M CuCl_2_·2H_2_O solution was added. The resulting mixture was subjected to sonication for 1.5 h, filtered, thoroughly washed with ethanol, and then dried for 3 h at 90 °C, yielding ∼0.2 g of the MWCNT-COO-Cu product. The recyclability and reusability of the catalyst were determined by separating it from the reaction mixture by employing a filtration process, washing it using ethanol, and then employing it in the oxidation reaction.

### 2.3 Preparation of the SILP and SCILL-SILP Catalytic Systems

The SILP catalytic systems were prepared by coating MWCNTs with a mixture of selected IL and NHPI via the solvent evaporation coating technique. The procedure involved mixing 0.025 g of IL and 0.027 g of NHPI with 0.025 g of MWCNTs in 5 mL of acetone. The mixture was sonicated for 30 min, followed by stirring at 1200 rpm for 3 h at room temperature. The solvent (acetone) was then evaporated using a vacuum evaporator while maintaining the bath temperature at 60 °C and gradually decreasing the pressure from 600 mbar to 300 mbar. The SCILL-SILP hybrid catalytic systems were prepared using the same technique, with the exception that MWCNTs were replaced with MWCNT-COO-Cu. The schematic illustration of the preparation of SILP and SCILL-SILP hybrid catalytic systems has been presented in Figure 1. The recyclability and reusability of the SILP and SCILL-SILP systems were performed by isolating the catalytic systems from the reaction mixtures using a filtration technique. The separated systems were thoroughly washed with hexane and then dried using a vacuum desiccator for 12 h. The dried catalysts were then used in the oxidation reactions.

**Figure 1:**
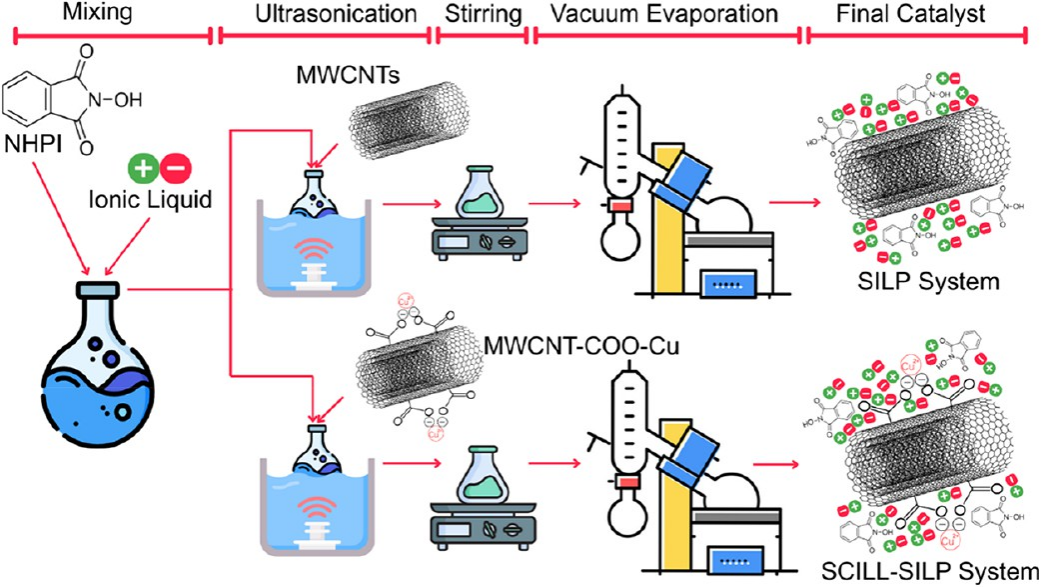
Schematic representation of the preparation of SILP and SCILL-SILP hybrid catalytic systems.

### 2.4 EB Oxidation

The oxidation reactions of EB were performed using a gasometric apparatus.^75^ The desired reaction mixture was placed in a 10 mL flask linked to a buret. Oxygen was introduced at atmospheric pressure, and the reaction mixture was maintained at 80 °C with continuous stirring at 1200 rpm using a magnetic stirrer. The volume of the consumed oxygen (O_2_) in mL was recorded from the buret, and the moles of O_2_ were determined using the following equation

1 $$n_{O_{2}}=\frac{V_{O_{2}}\times 273.15\times P}{101\;325\times 22\;415\times T}\;\left(mol\right)$$

In eq 1, *V*_O_2__ is the volume (mL) of consumed oxygen, and *P* and *T* represent the atmospheric pressure (Pa) and room temperature (K), respectively. Subsequently, the conversion of EB was determined using the following equation

2 $$\alpha =\frac{n_{O_{2}}}{n}\times 100\;\left(\%\right)$$

In eq 2, *n*_O_2__ denotes the moles (mol) of used O_2_, and *n* represents the moles (mol) of EB used.

The quantity of 1-phenylethyl hydroperoxide (PEHP) produced in each reaction mixture was determined using iodometric analysis.^76^ A sample weighing approximately 0.2 g (±0.0001 g) was taken from the reaction mixture shortly after the completion of the reaction and placed in a flask. To this, 20 mL of glacial acetic acid was added. The solution underwent carbon dioxide flushing to eliminate air, followed by the addition of approximately 2 g of sodium iodide. The flask was then sealed and placed in a dark place for 30 min to facilitate iodine formation. Subsequently, 20 mL of distilled water was added to the mixture, which was titrated using a standard 0.1 M sodium thiosulfate solution. The titration was performed employing a Brand Digital II apparatus with a measuring range of 25 mL and a 0.01 mL subdivision. The selectivity of the hydroperoxide was then determined using the following equation

3 $$S_{\mathrm{PEHP}}=\frac{n_{\mathrm{PEHP}}}{n_{O_{2}}}\times 100\;\left(\%\right)$$

In eq 3, *S*_PEHP_ represents the selectivity of the produced PEHP, *n*_PEHP_ is the quantity (mol) of PEHP in the reaction mixture, and *n*_O_2__ is the quantity (mol) of O_2_ consumed after the completion of the reaction.

PEHP is thermally unstable and readily decomposes into 1-phenylethanol (PEA) and/or acetophenone (AcPO). Therefore, it is imperative to reduce it to PEA before gas chromatography (GC) analysis. This reduction process involves the application of triethyl phosphite ((EtO)_3_P), and the utilized (EtO)_3_P undergoes oxidation, resulting in the formation of triethyl phosphate (EtO)_3_PO.^77^ Consequently, the amount of PEA measured through GC analysis is the combined result of the PEA produced in the reaction and the decomposed PEHP. The determination of the selectivity of PEA involves subtracting the selectivity of PEHP, as determined by iodometric analysis, from the selectivity of PEA acquired from GC analysis. GC analyses were performed using 4-methylanisole as an internal standard.

### 2.5 Safety Precautions

The liquid-phase aerobic oxidation reactions of EB, as well as the product sampling for analyses and catalyst separations, were performed under a fume hood with appropriate personal protective equipment (PPE). This was crucial due to the potential hazards associated with EB and its oxidation products, especially with the PEHP, a thermally unstable organic peroxide. Even though the reactions were performed at atmospheric pressure, the mixture of the produced PEHP and molecular oxygen can still create a favorable environment for the formation of a fuel-oxidant mixture, which may lead to an explosion under certain circumstances. Consequently, it is necessary to control the reaction temperature and pressure and eliminate any ignition sources. Therefore, we strictly followed standard safety protocols throughout the experiments to minimize any associated risks. After each reaction, samples were carefully collected, catalysts were separated, and the remaining reaction mixtures were diluted and safely disposed of following the standard laboratory procedures.

### 2.6 Analytical Methods

Gas chromatography (GC) analyses were performed using a Shimadzu GC-2010 Plus gas chromatograph from Kyoto, Japan. The instrument was equipped with an FID (Flame Ionization Detector) and ZB-5HT column (29.5 m in length and 0.25 mm in internal diameter). The morphologies of the synthesized catalytic systems were analyzed by employing the transmission electron microscope (TEM) of the Thermo Fisher Scientific TecnaiG2 F20 X-TWIN/FEI. The thermal stability and the leaching of the hybrid catalysts’ components were investigated using thermogravimetric analyses (TGA) with the TGA/DSC STAR system analyzer from Mettler Toledo. The temperature range was from 25–800 °C with a heating rate of 10 °C min^–1^ with a constant nitrogen flow of 60 mL min^–1^. Fourier-transform infrared (FTIR) spectra were acquired employing a Spectrum Two Spectrometer (PerkinElmer) with a diamond crystal attenuated total reflectance (ATR) accessory. Spectra were acquired from 4000 to 400 cm^–1^ with a resolution of 4 cm^–1^ and averaged across four scans.

## 3 Results and Discussion

In this study, novel SILP and SCILL-SILP hybrid catalytic systems were developed. SILP systems were synthesized by coating NHPI dissolved in IL onto pristine MWCNTs, whereas SCILL-SILP hybrid catalytic systems were synthesized by coating NHPI dissolved in IL onto Cu­(II) functionalized carboxylated MWCNTs (Figure 2). To develop more stable and active SILP and SCILL-SILP hybrid catalytic systems, ILs with various anions and cations have been utilized, i.e., [emim], [bmim], [omim], or [C_10_mim] cations and [Cl], [Br], [BF_4_], [PF_6_], [OcOSO_3_], or [NTf_2_] anions. The recyclability and reusability of the SILP and SCILL-SILP catalytic systems have also been thoroughly examined. In addition, leaching tests were conducted for both catalytic systems.

**Figure 2:**
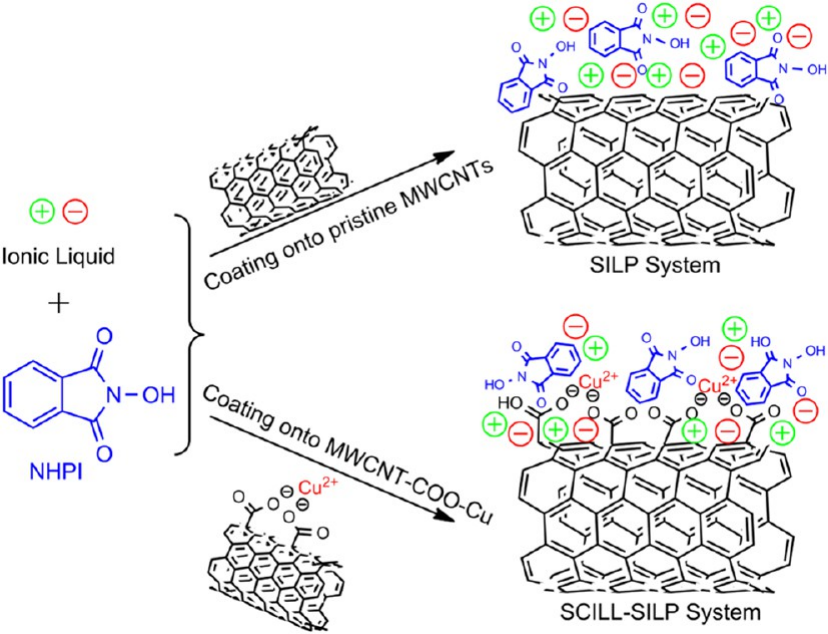
SILP and SCILL-SILP hybrid catalytic systems.

As an example, the produced [bmim]­[OcOSO_3_]-based SILP and SCILL-SILP hybrid catalytic systems were characterized by employing TEM, ATR-FTIR, and TGA and DTG analyses. The smooth and defect-free nanotubes having a 20–40 nm outer diameter with no impurities can be observed from the TEM image of pristine MWCNTs (Figure 3a). On the other hand, the nonuniform thin layer of dissolved NHPI in IL over the surface of pristine MWCNTs (SILP system) and Cu­(II) functionalized CNTs (SCILL-SILP hybrid catalytic system) can be observed from the TEM images in Figure 3b,c, respectively. It was already demonstrated in our previous paper^74^ that coating the mixture of dissolved NHPI in IL onto the surface of Cu­(II) functionalized MWCNTs significantly reduced the BET surface area, total pore volume, and total pore area.

**Figure 3:**
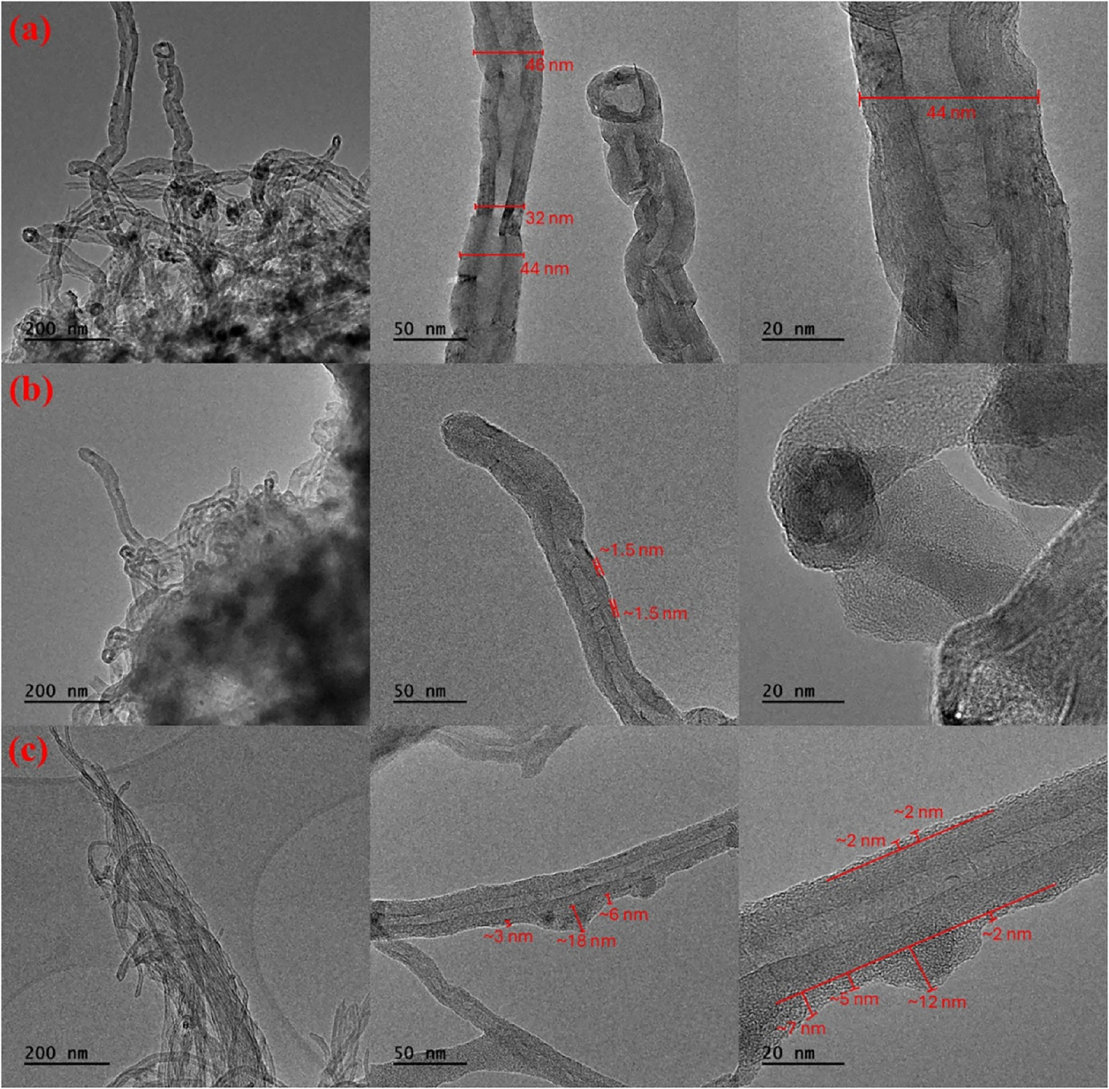
TEM image of (a) Pristine MWCNTs, (b) [bmim]­[OcOSO_3_]-based SILP system, (c) [bmim]­[OcOSO_3_]-based SCILL-SILP system.

The ATR-FTIR spectra of the [bmim]­[OcOSO_3_]-based SILP and SCILL-SILP systems, as well as their components, were acquired and compared (Figure 4). As can be observed in Figure 4a, the carboxylated MWCNTs (MWCNT-COOH) exhibit a broad absorption band at ∼3380 cm^–1^, which could be assigned to the O–H stretching vibration of surface carboxyl groups. Peaks at ∼2923 and ∼2853 cm^–1^ are attributed to the asymmetric and symmetric stretching of the methylene (−CH_2_) groups, typically associated with surface defects.^79^ The peaks at ∼1704 cm^–1^ could be assigned to the C=O stretching of the carboxyl group. The band at ∼1611 could be assigned to the C=C stretching in the graphitic framework, while the peak at ∼1577 cm^–1^ could be related to the carboxylate anion stretching vibrations.^80^ After Cu­(II) functionalization, the corresponding MWCNT-COO-Cu (Figure 4a) demonstrated a reduction in the intensities of the O–H (∼3380 cm^–1^), C=O (∼1702) and the carboxylate anion stretching bands at ∼1577 cm^–1^, indicating successful coordination of Cu­(II) to the carboxylate groups. The divalent Cu­(II) ions could have been immobilized as (MWCNT-COO)_2_Cu or MWCNT-COOCuCl.

**Figure 4:**
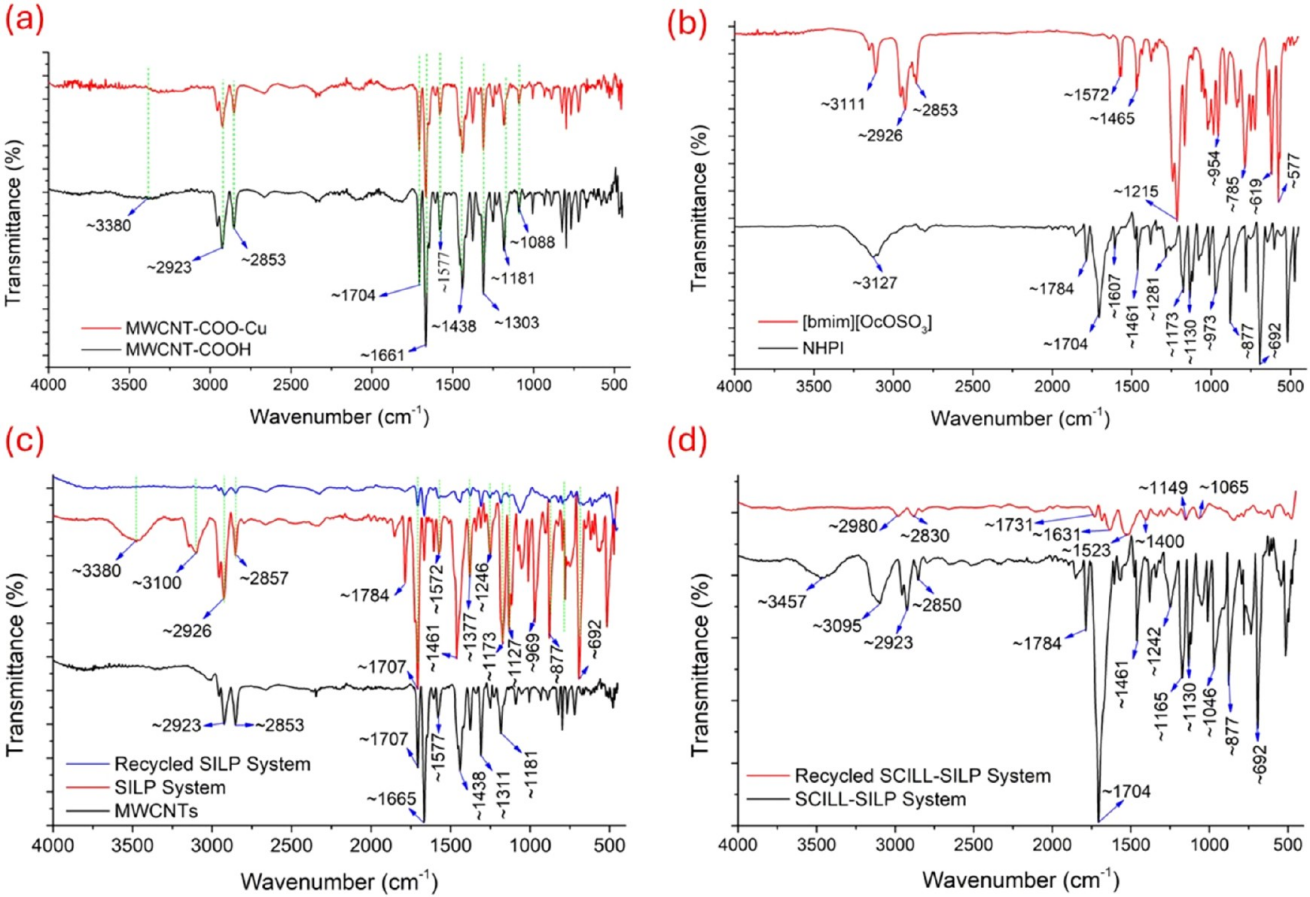
FTIR spectra of (a) MWCNT–COOH and MWCNT-COO-Cu, (b) NHPI and [bmim]­[OcOSO_3_], (c) MWCNTs, fresh and recycled [bmim]­[OcOSO_3_]-based SILP systems, and (d) fresh and recycled [bmim]­[OcOSO_3_]-based SCILL-SILP systems.

The ATR-FTIR spectrum of the MWCNTs (Figure 4c) depicts that it has surface defects and oxygenated functional groups, as its spectrum exhibited similar to the carboxylated MWCNTs. Upon coating the NHPI dissolved [bmim]­[OcOSO_3_] onto the surface of MWCNTs, the resulting SILP system (Figure 4c) exhibited broad peaks at 3380 cm^–1^, which could be related to the sorbed water and/or the oxygen-containing functional groups on the MWCNTs. The absorption bands at ∼3100, 1784, and 1704 cm^–1^ could be attributed to the N–OH stretching, and the symmetric and asymmetric C=O stretching vibrations of the carbonyl groups in the NHPI.^81^

The characteristics ATR-FTIR spectrum of [bmim]­[OcOSO_3_] IL, as can be observed in Figure 4b, includes a peak at ∼3111 cm^–1^, attributed to C–H stretching in the imidazolium ring. The peaks at ∼2926 and ∼2853 cm^–1^ correspond to asymmetric and symmetric stretching vibrations of C–H in the alkyl chains. The band at ∼1571 cm^–1^ could be attributed to C=C and C=N stretching vibrations in the imidazolium ring, while the band at ∼1467 cm^–1^ is attributed to CH_2_ bending vibrations from the alkyl chains of the [bmim] cation and [OcOSO_3_] anion. The strong and sharp absorption band at 1215 cm^–1^ could be assigned to the S=O stretching in the sulfate anion.^82^ However, all these peaks in the SILP system have overlapped with the absorption bands of NHPI and MWCNTs. The ATR-FTIR spectrum of the SCILL-SILP system (Figure 4d) has also well-pronounced peaks at ∼3095, ∼1784, and ∼1704 cm^–1^, indicating the presence of NHPI; however, the characteristic IR bands of [bmim]­[OcOSO_3_] are again obscured due to overlapping.

The catalytic activities of the produced SILP and SCILL-SILP hybrid catalytic systems were examined in the solvent-free aerobic oxidation of EB, which served as a model hydrocarbon. All reactions were performed at 80 °C, 0.1 MPa, and 1200 rpm for 6 h. The conversion of EB was determined by measuring the quantity of oxygen consumed, and the composition of the reaction mixture was analyzed using both iodometric and GC methods. The identified products are PEHP, PEA, AcPO, benzaldehyde (BA), and benzoic acid (BzA), as illustrated in Scheme 2.

**Scheme 2:**
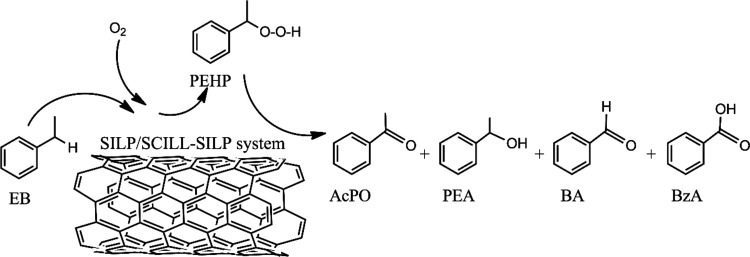
Oxidation Products of EB

### 3.1 Catalytic Activities of the SILP Systems

SILP systems were produced to combine the known catalytic activities of CNTs, NHPI, and ILs in the free-radical liquid-phase oxidation of hydrocarbons. Their catalytic activities in the solvent-free oxidation of EB have been presented in Figure 5. It was observed that both NHPI and MWCNTs slightly increased the EB conversion. The lower selectivity of PEHP in the presence of MWCNTs confirmed its activity in the decomposition of hydroperoxides. When NHPI was added with CNTs, the selectivity of PEHP increased significantly. This enhancement could be attributed to the known higher selectivity of hydroperoxides in the presence of NHPI.^83,84^

**Figure 5:**
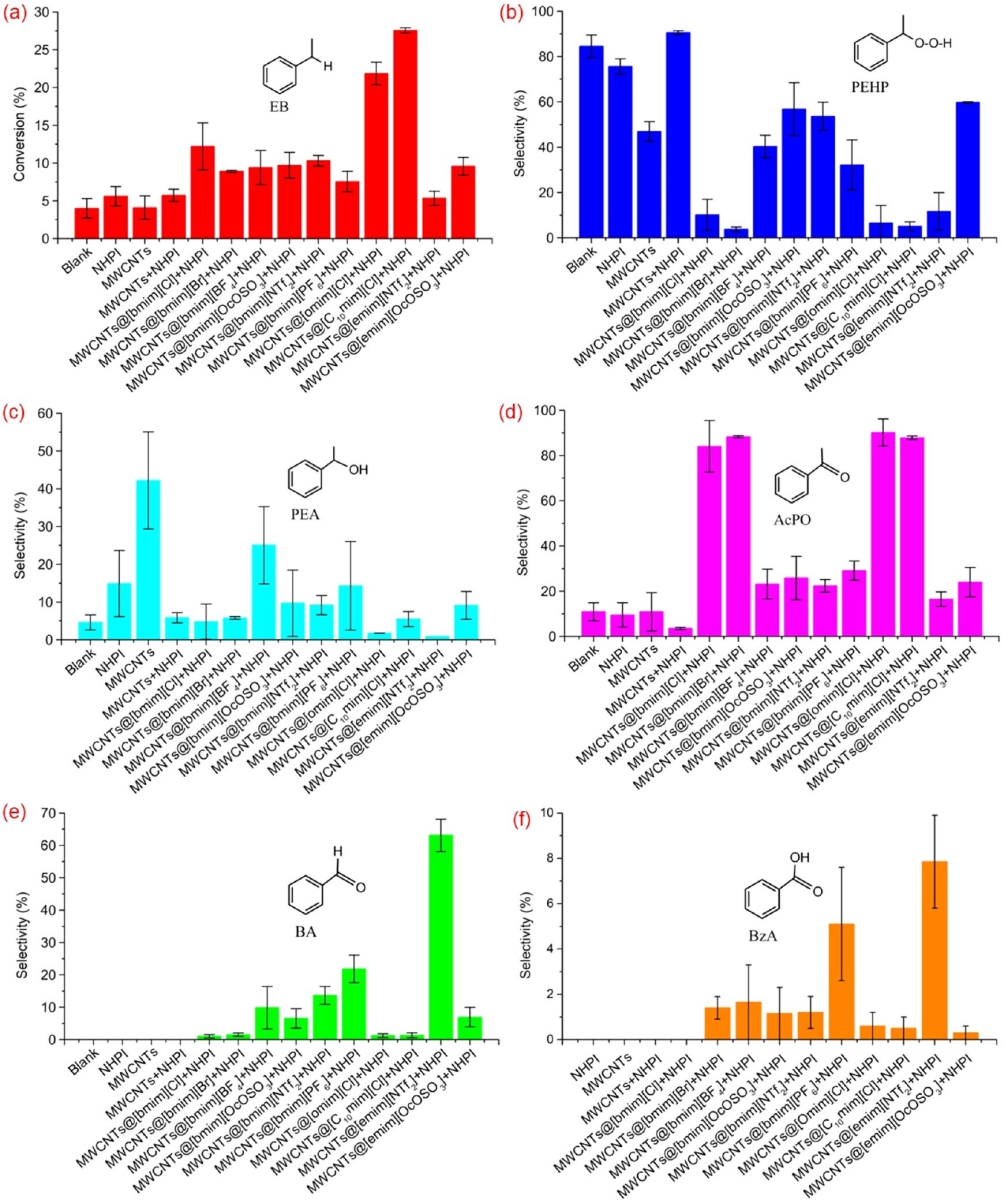
Catalytic activities of SILP systems: (a) EB conversion (%), (b) PEHP selectivity (%), (c) PEA selectivity (%), (d) AcPO selectivity (%), (e) BA selectivity (%), and (f) BzA selectivity (%). Reaction conditions: EB = 16.3 mmol, MWCNTs = 0.025 g, NHPI = 0.027 g, IL = 0.025 g, AIBN = 0.186 mol %, 80 °C, 0.1 MPa, 6 h.

All the [bmim] cationic IL-based SILP systems exhibited higher catalytic activities in the oxidation reactions compared to pristine MWCNTs and MWCNTs/NHPI. The observed effects, among others, could be a result of IL activity in catalyzing hydroperoxide decomposition, NHPI-IL interaction enhancing PINO formation, reagents and NHPI solubility in the IL layer, ILs polarity and viscosity, as well as ILs and NHPI leaching from the MWCNTs surface.

The highest conversion of EB (14.4%) was achieved for the SILP system based on [bmim]­[Cl], with the highest selectivity to AcPO (92.1%). The higher activity of the SILP system, MWCNTs@[bmim]­[Cl], compared to MWCNTs@[bmim]­[Br] aligns with previous results on the influence of halide ILs in NHPI-catalyzed hydrocarbon oxidation reactions reported by Talik et al.^60^ They discovered that the activity of ILs containing 1-alkyl-3-methylimidazolium cation and halide anion, [Rmim]­[X], in NHPI-catalyzed oxidation reactions was decreased in the order of [Cl] > [Br] > [I]. Their research revealed that there is an interaction between the halide anion in the IL and the hydrogen atom in the –NOH group of NHPI (Figure 6), which can significantly impact the reaction rate. This interaction may weaken the NO–H bond, facilitating PINO generation. The strength of the interaction can be correlated with the acidity of the HX acids, as determined by the specific anions [X], with their strength increasing in the series [Cl] < [Br] < [I]. At the same time, the weaker the acid, the stronger its conjugate base, indicating that the –NOH–[Cl] interactions are stronger than the –NOH–[Br] and –NOH–[I] interactions, resulting in faster PINO generation and ultimately improving the reaction rate.

**Figure 6:**
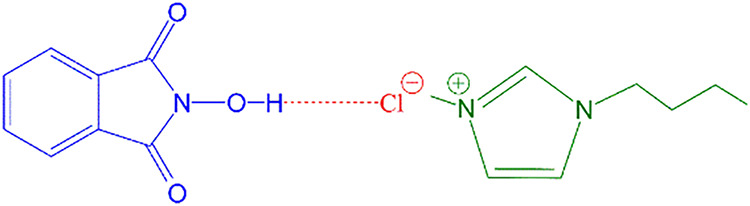
Interaction between NHPI and [bmim]­[Cl]. Adapted with permission from ref 60. Copyright 2021 Wiley-VCH.

The SILP systems produced using [bmim]­[NTf_2_] and [bmim]­[OcOSO_3_] demonstrated higher selectivity to PEHP. Despite their limited hydroperoxide decomposition activity, they demonstrated rather significant catalytic activity, potentially due to their better oxygen solubility. The oxygen solubility in the [bmim] cationic ILs decreases in the following order: NTf_2_ > PF_6_ ∼ OcOSO_3_ > BF_4_.^85^ The oxygen solubility in [bmim]­[PF_6_] is almost similar to that in [bmim]­[OcOSO_3_]; however, the NHPI displayed very little solubility in this particular IL.

The impact of the cation structure of the IL on the catalytic activity of the SILP system has also been investigated. It was found that when the number of carbon atoms in the alkyl group of the cations in the IL of the SILP system increased, such as in [omim]­[Cl] and [C_10_mim], the conversion of EB also increased with enhanced selectivity toward AcPO. It is known that enhancing the lipophilicity of cations in the ILs leads to a higher solubility of hydrocarbons and oxygen in the ILs, as well as the solubility of ILs in nonpolar systems.^60,72,86^ However, this could potentially cause the undesired leaching of IL from the surface of MWCNTs.

To make a comparison, when the [bmim] cation of [OcOSO_3_] and [NTf_2_] was replaced with the [emim] cation to make a less lipophilic SILP system, the conversion of EB was drastically decreased for the SILP system composed of [emim]­[NTf_2_] with more selectivity toward BA (61.0%). BA formed as a result of the β–C–C cleavage in the 1-phenylethyloxy radical.^87,88^ This may represent the robust impact of [emim]­[NTf_2_] on the cleavage of the alkoxy radical. The [emim]­[NTf_2_]-based SILP system had the highest selectivity toward BA, followed by [bmim]­[PF_6_] and [bmim]­[BF_4_]. The SILP system based on [emim]­[OcOSO_3_] still had a higher selectivity toward hydroperoxide, with the conversion of EB comparable to that of the [bmim]­[OcOSO_3_]-based SILP system.

### 3.2 Catalytic Activities of SCILL-SILP Hybrid Systems

The SCILL-SILP hybrid catalytic systems were developed to synergistically use the known catalytic activities of CNTs, NHPI, ILs, and copper­(II) salts for the oxidation of hydrocarbons in the liquid phase using oxygen as an oxidant. The results of EB oxidation in the presence of SCILL-SILP systems are presented in Figure 7. For comparison, the catalytic activity of MWCNT-COO-Cu was also investigated in the presence of NHPI as a cocatalyst.

**Figure 7:**
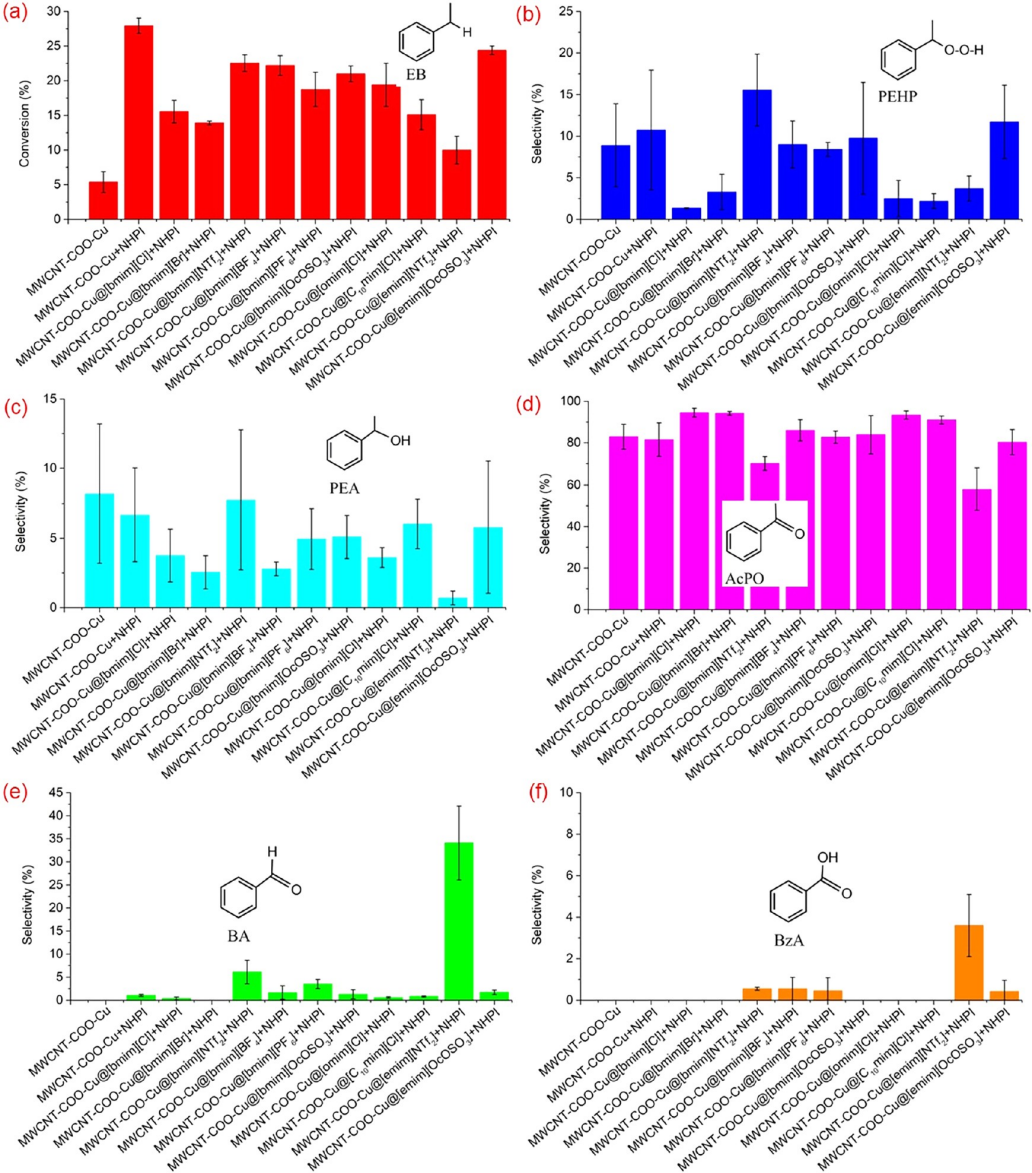
Catalytic activities of SCILL-SILP systems: (a) EB conversion (%), (b) PEHP selectivity (%), (c) PEA selectivity (%), (d) AcPO selectivity (%), (e) BA selectivity (%), and (f) BzA selectivity (%). Reaction conditions: EB = 16.3 mmol, MWCNT-COO-Cu = 0.025 g, NHPI = 0.027 g, IL = 0.025 g, AIBN = 0.186 mol %, 80 °C, 0.1 MPa, 6 h.

It was observed that the catalytic activity of Cu­(II)-functionalized CNTs increased significantly compared to MWCNTs when subjected to similar reaction conditions; the EB conversion increased from ∼5.7 ± 0.8% to ∼27.9 ± 1.1%. The catalytic activity of the MWCNT-COO-Cu/NHPI system is due to the synergistic effect of Cu­(II)-functionalized CNTs and NHPI. Transition metal compounds, including Cu­(II) compounds, are known to enhance the decomposition of hydroperoxide into the corresponding alcohol and ketone, consequently enhancing the rate of the oxidation reaction.^89–92^ MWCNT-COO-Cu also accelerated the formation of PINO radicals from NHPI. Furthermore, the literature has reported that the distribution of products in hydrocarbon oxidation reactions is dependent upon the specific type of transition metal salt employed. The presence of Cu­(II) salts produced a higher ratio of ketone to alcohol compared to Co­(II) and Mn­(II) because of differences in their redox potentials.^59^ Therefore, the ratio of ketone to alcohol for the MWCNT-COO-Cu/NHPI system was also high.

The catalytic activities of the SCILL-SILP hybrid catalytic systems were lower compared to the MWCNT-COO-Cu/NHPI catalyst and were dependent on the type of IL used. Among the [bmim] cationic ILs-based SCILL-SILP systems, the obtained EB conversion decreases in the following order of anions: [NTf_2_] > [BF_4_] ≈ [PF_6_] ≈ [OcOSO_3_] > [Cl] > [Br]. The solubility of oxygen in [bmim]-based ILs at 50 °C (in mmol·dm^–3^·atm^–1^) follows the order: [NTf_2_] (3.75) > [PF_6_] (2.67) > [OcOSO_3_] (2.62) > [BF_4_] (1.49),^93–95^ whereas their viscosities at 30 °C (in mPa·s) follow the opposite trend: [NTf_2_] (41.24) < [bmim]­[BF_4_] (75) < [bmim]­[PF_6_] (182) < [bmim]­[OcOSO_3_] (447.9) < [bmim]­[Cl] (11,000).^96–99^ Consequently, the catalytic performance of [bmim] cation-based SCILL-SILP system is influenced by the oxygen solubility and viscosity of the IL. Among the studied SCILL-SILP systems, the [bmim]­[NTf_2_] based system demonstrated higher catalytic activity in the oxidation of EB, owing to its high oxygen solubility and low viscosity. All the SCILL-SILP-based systems demonstrated higher selectivity toward AcPO in comparison to their respective SILP systems.

The obtained results demonstrated that the availability of Cu­(II) played a significant role and could be influenced, among others, by the IL viscosity. The obtained EB conversion with remarkable AcPO selectivity for the [bmim]­[Cl]-based SCILL-SILP and SILP systems was approximately identical (Figures 7 and 5), which suggested that the highly viscous [bmim]­[Cl] limited the availability of Cu­(II). Additionally, the high selectivity to AcPO could also be attributed to the solvent cage effect of highly viscous [bmim]­[Cl]. The coated IL acted as a cage, confining the produced alkoxy radicals and restricting their ability to pass through the cage and react with EB to produce PEA. Therefore, the β-scission reaction inside the IL-solvent cage is favored.^100^

The effect of the cation lipophilicity in the SCILL-SILP system was also investigated. Increasing the lipophilicity of the cation in [Cl]-based ILs by using [omim] and [C_10_mim] in the SCILL-SILP system initially enhanced the EB conversion for the [omim]­[Cl]-based system; however, a further increase with [C_10_mim]­[Cl] decreased the EB conversion (Figure 7). The activity of the [C_10_mim]­[Cl]-based system remained comparable to that of the [bmim]­[Cl]-based SCILL-SILP system. Similarly, both systems demonstrated higher selectivity toward AcPO; however, their overall catalytic activity was lower compared to the corresponding SILP systems. This could be due to their interaction with the MWCNT-COO-Cu. The lipophilic ILs leached more readily from the SCILL-SILP systems; this leaching may facilitate interactions between the strongly coordinating [Cl] anions in the ILs and the immobilized Cu­(II) species on the MWCNTs. Such interactions may not only reduce the availability of Cu­(II) active sites but also limit the positive interaction of [Cl] anions with the –N–OH group in NHPI, thereby affecting the rate of PINO radical generation. Consequently, these effects reduced the overall catalytic activity of the [omim]­[Cl] and [C_10_mim]­[Cl]-based SCILL-SILP systems compared to their corresponding SILP systems.

### 3.3 Recyclability of SILP Systems

The recyclability and reusability of SILP catalytic systems were investigated, and the results are presented in Figure 8. The SILP catalytic systems were separated from the reaction mixture via the filtration process and washed with hexane. The dried catalysts were subsequently used to oxidize a fresh batch of EB. The SILP system based on [bmim]­[OcOSO_3_] IL was demonstrated to be the most stable compared to the other IL-based SILP systems in this study. This stability could be associated with the amphiphilic nature of the [OcOSO_3_] anion, which is composed of a hydrophilic sulfate head and hydrophobic octyl tail. The dual character of this specific anion may facilitate interactions with the polar and nonpolar components in the SILP system, as well as with the reaction mixture. The hydrophobic octyl tail may facilitate the interaction of this specific IL with the hydrophobic MWCNTs. At the same time, this specific IL could also facilitate better dissolution of NHPI due to its partially hydrophilic nature. It was observed that the system’s reusability decreased as the lipophilicity of the cation increased. For instance, by substituting the [bmim] cation in the [Cl]-based anionic IL with [omim] and [C_10_mim], the conversion increased substantially for the fresh samples. Nevertheless, their catalytic activities decreased drastically in the subsequent cycles. It could have been due to the undesirable leaching of IL from the catalyst surface.

**Figure 8:**
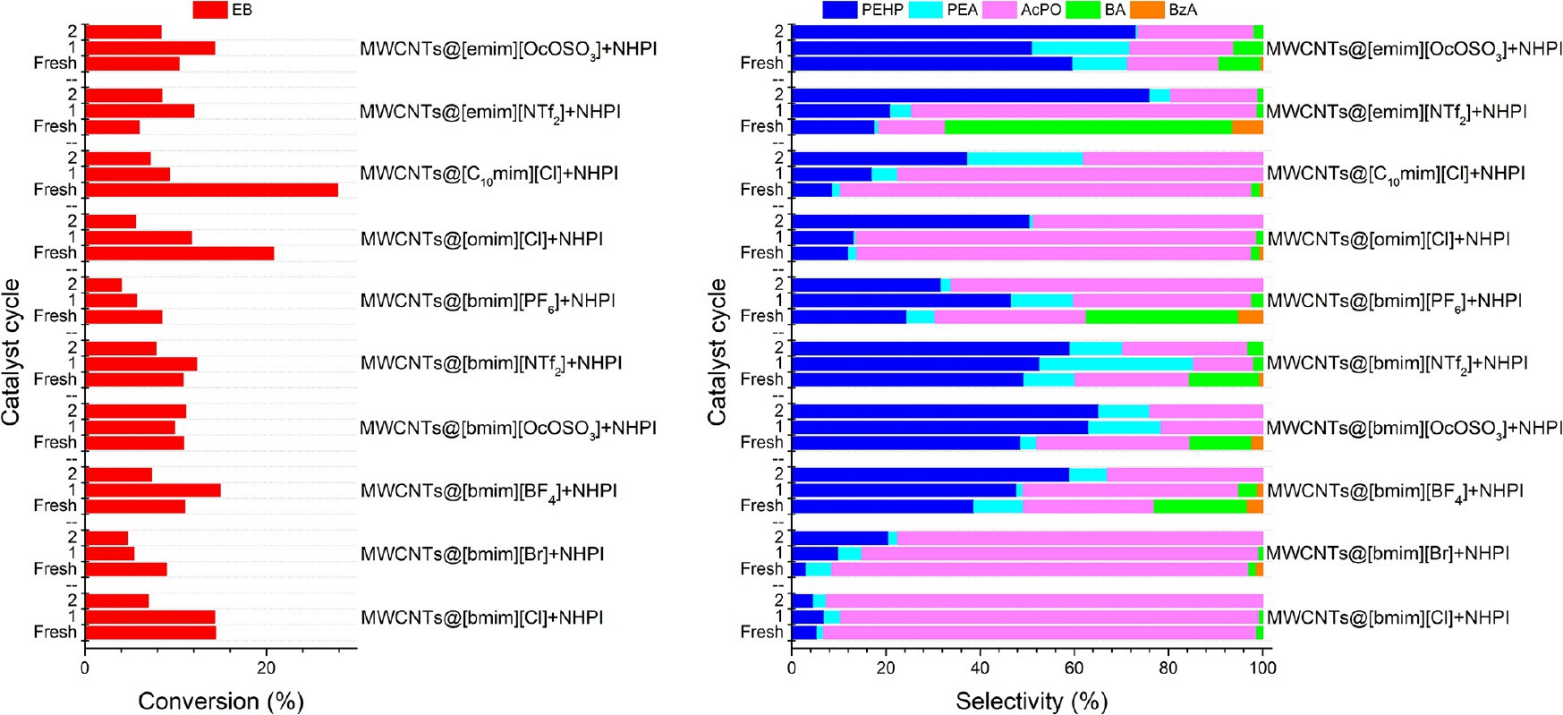
Recyclability and reusability of the SILP systems. Reaction conditions: EB = 16.3 mmol, MWCNTs = 0.025 g, NHPI = 0.027 g, IL = 0.025 g, AIBN = 0.186 mol %, 80 °C, 0.1 MPa, 6 h.

To examine the reusability of the SILP system based on [bmim]­[OcOSO_3_], a leaching test was performed. A fresh [bmim]­[OcOSO_3_]-based SILP system was prepared and applied in the oxidation reaction. After 30 min, the SILP system was isolated from the reaction mixture, and the reaction effluent was allowed to be run to complete the specified reaction time. For comparison, reactions were also performed employing [bmim]­[OcOSO_3_], [bmim]­[OcOSO_3_] + NHPI, and MWCNTs + [bmim]­[OcOSO_3_] + NHPI. The results are shown in Table 1 and Figure 9.

**Figure 9:**
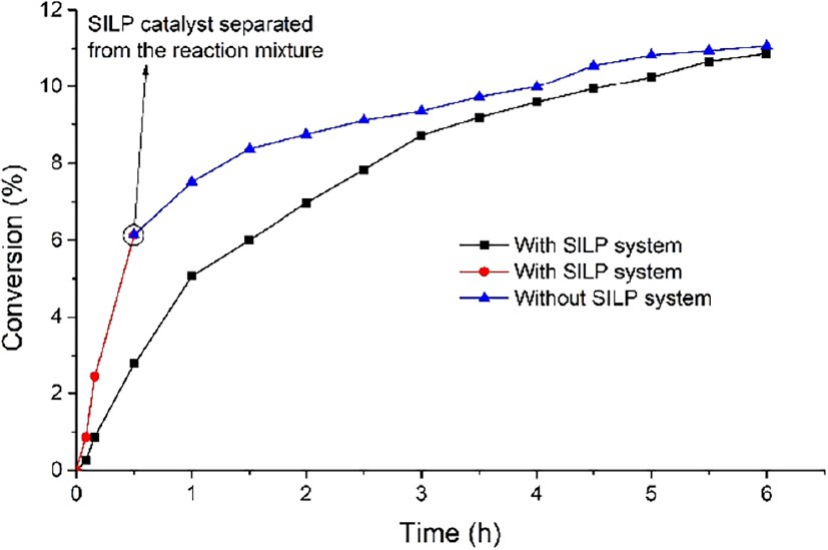
Comparison of EB conversion (%) as a function of time (h) for the SILP catalytic system with the system in which the SILP was separated during the reaction and the reaction mixture was allowed to proceed and complete the specified reaction time.

**Table 1:**
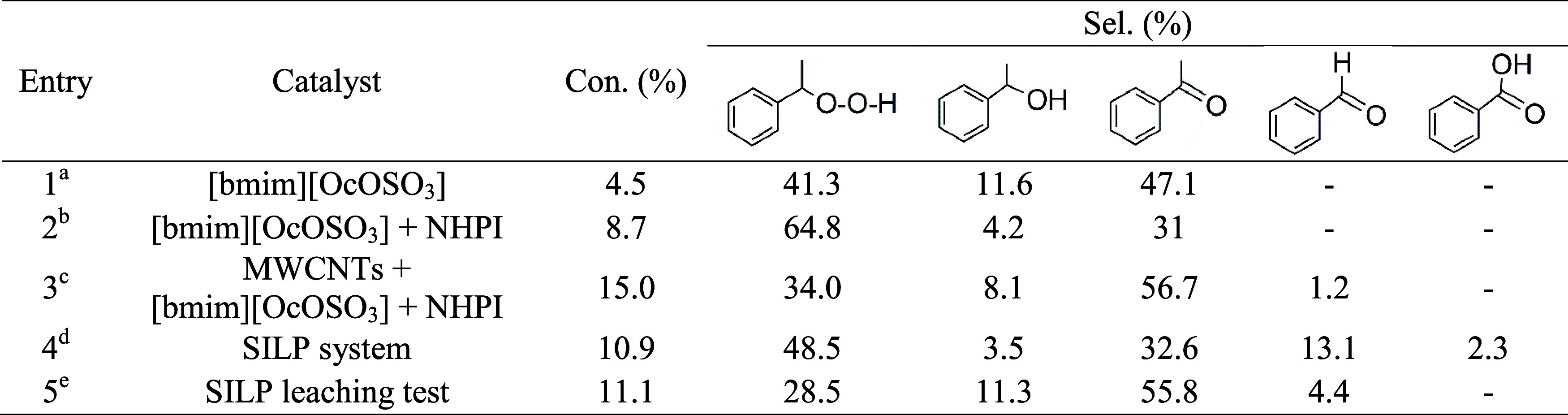
Leaching Test for the SILP System^f^

a 0.025 g IL.

b 0.025 g IL and 0.027 g of NHPI.

c 0.025 g MWCNTs, 0.025 g IL, and 0.027 g NHPI.

d SILP system prepared by coating the mixture of [bmim]­[OcOSO_3_] IL (0.025 g) and NHPI (0.027 g) over MWCNTs (0.025 g)

e SILP system was separated from the reaction mixture during the reaction, and the effluent was allowed to proceed and complete the reaction time.

f Reaction conditions: EB = 16.3 mmol, AIBN = 0.186 mol %, 80 °C, 0.1 MPa, 6 h.

It was observed that, after the separation of the SILP system from the reaction mixture, the reaction rate decreased slightly. The conversions obtained after 6 h in reactions carried out in the presence of SILP and after SILP separation were approximately identical, indicating the undesirable leaching of the catalysts. The product composition suggests that mainly IL was leached out from the surface of MWCNTs. The selectivity to PEHP and AcPO achieved following the separation of the SILP system (Table 1, entry 5) was comparable to that of the reaction run employing pristine [bmim]­[OcOSO_3_] (Table 1, entry 1).

The leaching of the mixture of IL and NHPI from the surface of MWCNTs was also confirmed by TGA and DTG analyses performed for both the fresh and recycled SILP systems after the second cycle (Figure 10). As shown in Figure 10a, the fresh SILP system lost weight in two major thermal decomposition stages. In the first stage, 32.76% weight loss occurred at a higher thermal decomposition rate at a temperature of 241 °C, which could be attributed to the decomposition of NHPI.^101^ In the second stage, 31.13% weight was observed with a higher thermal decomposition rate at 310 °C, which could be assigned to the decomposition of [bmim]­[OcOSO_3_].^17^ On the other hand, the recycled SILP system (Figure 10b) exhibited a reduced thermal degradation profile in two stages.^102,103^ The first stage exhibited 20.29% weight loss, while the second stage showed 7.25% weight loss, with the maximum decomposition rate at 183.71 °C, confirming the partial leaching of NHPI and IL from the SILP system upon recycling. The FTIR spectrum of the recycled SILP system (Figure 4c) further confirmed the undesirable partial leaching of NHPI and IL, as the obtained IR bands have reduced and/or negligible intensities.

**Figure 10:**
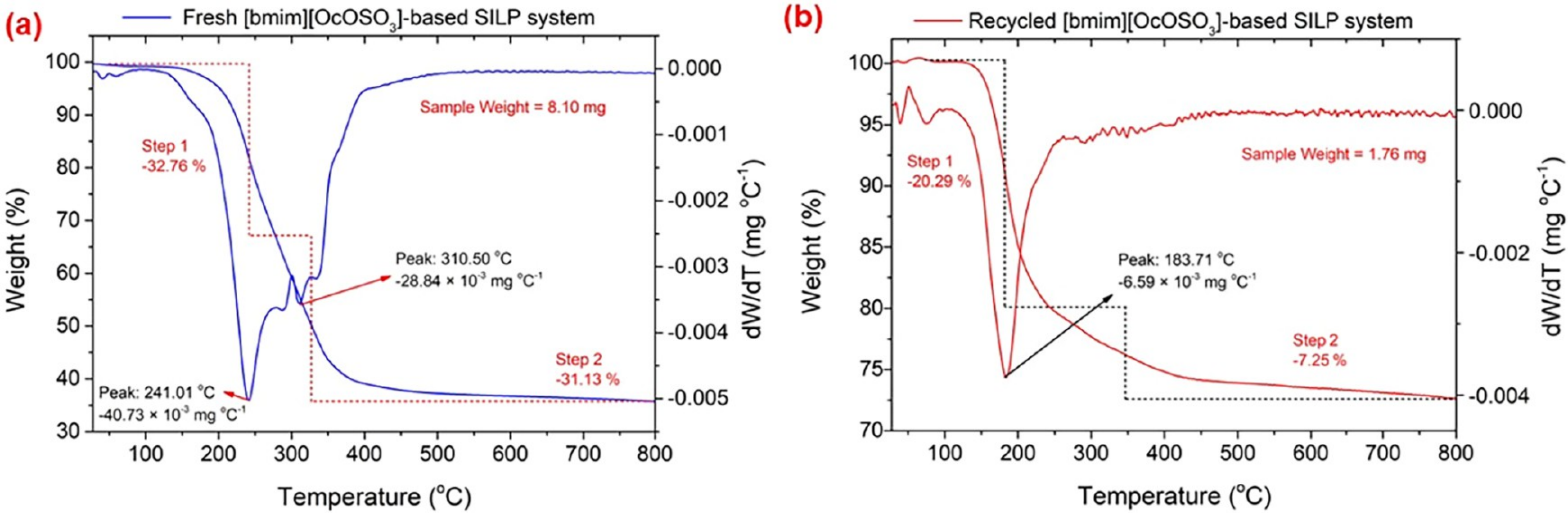
TGA and DTG curves, obtained for both the (a) fresh and (b) recycled SILP catalytic systems after the second cycle.

### 3.4 Recyclability and Reusability of SCILL-SILP Hybrid Catalytic Systems

An investigation was conducted to determine whether the SCILL-SILP catalytic systems were recyclable and reusable, and the results have been presented in Figure 11. Unfortunately, the catalytic activities of all SCILL-SILP systems decreased significantly after recovery and reuse. The results demonstrated that the decrease in the catalytic activities is not only associated with the leaching of the mixture of ILs and NHPI but also with the leaching of Cu­(II) from the MWCNT-COO-Cu. This is evident from the observation that the catalytic activity of Cu­(II) also dropped substantially in the second cycle while exhibiting higher selectivity toward hydroperoxides (Figure 11). The catalytic activity of the [bmim]­[OcOSO_3_]-based system was more stable compared to the other IL-based SCILL-SILP systems, which may again be attributed to the amphiphilic nature of the [OcOSO_3_] anion. The polar sulfate group in the [OcOSO_3_] anion may interact with the Cu­(II) sites on the functionalized MWCNTs in the SCILL-SILP systems, while the hydrophilic nature of this IL could also facilitate better dissolution of NHPI.

**Figure 11:**
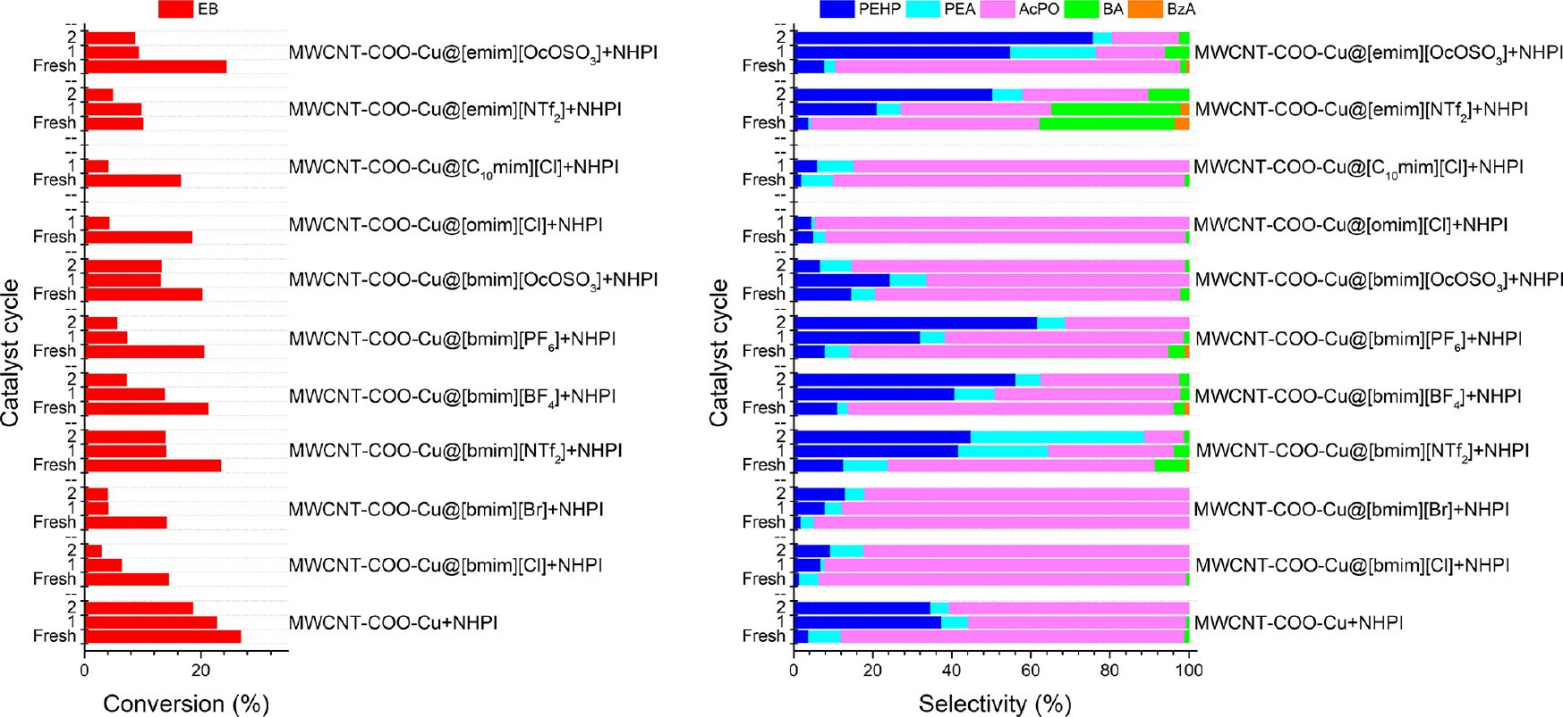
Recyclability and reusability of the SCILL-SILP system. Reaction conditions: EB = 16.3 mmol, MWCNTs = 0.025 g, NHPI = 0.027 g, IL = 0.025 g, AIBN = 0.186 mol %, 80 °C, 0.1 MPa, 6 h.

To examine the leaching of the mixture of IL and NHPI from the surface of the catalyst, a test was performed employing a more stable SCILL-SILP catalytic system, which was the [bmim]­[OcOSO_3_]-based system, and the results have been tabulated in Table 2. The SCILL-SILP system was isolated shortly after running the reaction (30 min), and the reaction mixture was allowed to complete the reaction time, as shown in Figure 12. It can be observed that the rate of reaction significantly decreased after the SCILL-SILP system separation, and the final conversion was almost half of that attained in the presence of the SCILL-SILP system, demonstrating that only a portion of the NHPI and IL has been leached out from the surface of MWCNT-COO-Cu.

**Figure 12:**
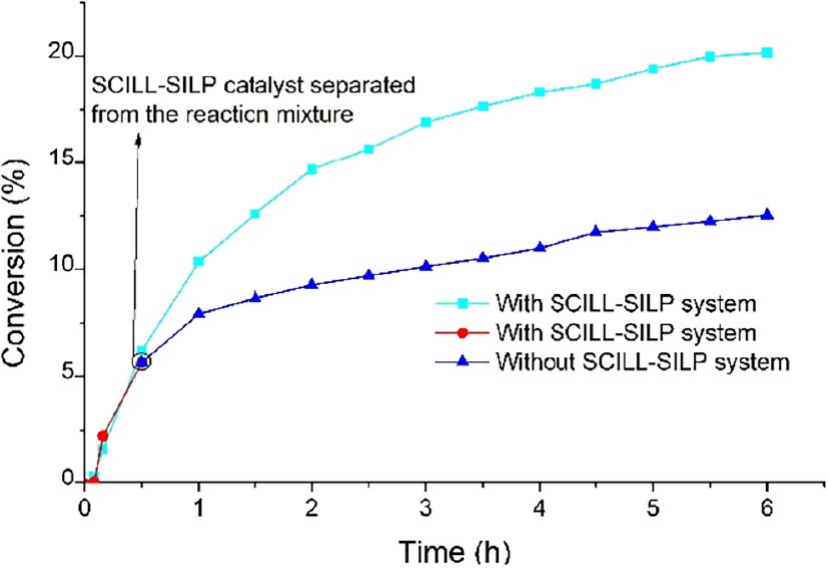
Comparison of EB conversion (%) as a function of time (h) for the SCILL-SILP catalytic system with the system in which the SCILL-SILP was separated during the reaction and the reaction mixture was allowed to proceed and complete the specified reaction time.

**Table 2:**
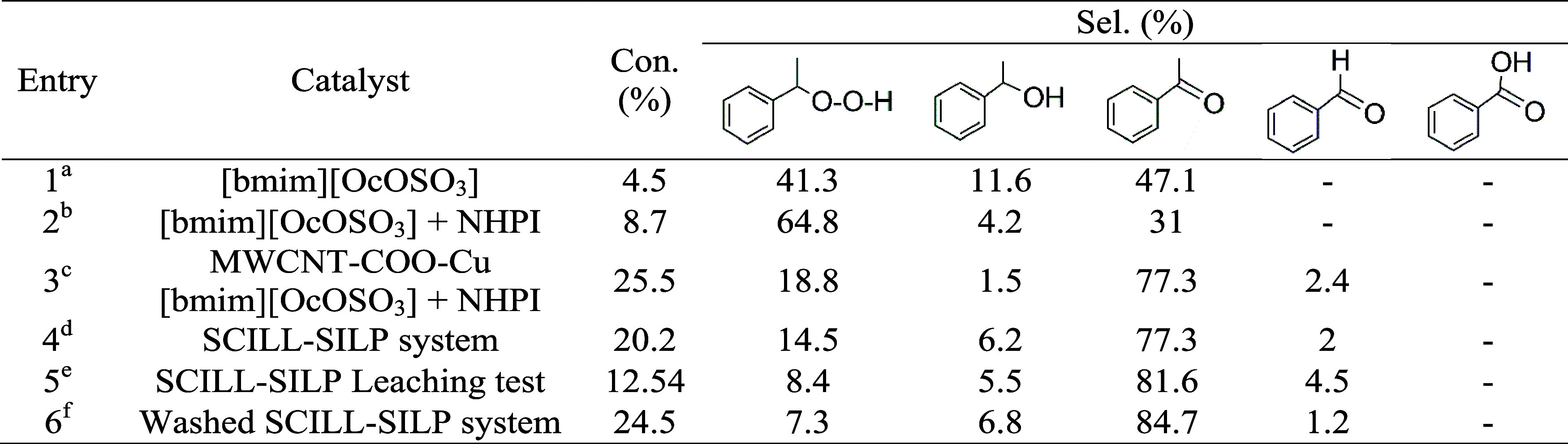
Leaching Test for the SCILL-SILP System^g^

a 0.025 g IL.

b 0.025 g IL and 0.027 g of NHPI added separately to EB.

c Mixture of 0.025 g MWCNTs, 0.025 g IL and 0.027 g NHPI added separately to EB.

d SCILL-SILP system prepared by coating the mixture of IL [bmim]­[OcOSO_3_] (0.025 g) and NHPI (0.027 g) over MWCNT-COOH-Cu (0.025 g).

e SCILL-SILP system was separated from the reaction mixture during the reaction and the effluent was allowed to proceed and complete the reaction time.

f Fresh SCILL-SILP system was washed with hexane before applying in the oxidation reaction.

g Reaction conditions: EB = 16.3 mmol, AIBN = 0.186 mol %, 80 °C, 0.1 MPa, 6 h.

The leaching of IL and NHPI could be associated with the fact that as the reaction time proceeded, more polar products formed, and the solubility of IL and NHPI in the reaction mixture increased. To confirm the statement, the [bmim]­[OcOSO_3_]-based SCILL-SILP system was washed thoroughly with a nonpolar solvent (hexane) before being applied to the oxidation reaction (Table 2, entry 6). It is worth noting that washing the SCILL-SILP system with the nonpolar solvent does not affect its activity, and the conversion of EB (24.5%) and the selectivity toward AcPO (84.7%) were even higher.

TGA and DTG analyses were performed for the fresh and recycled SCILL-SILP systems (Figure 13) to further analyze the undesirable leaching of NHPI and IL. It can be observed from Figure 13a that the fresh SCILL-SILP system lost weight in two major thermal decomposition stages. In the first stage, 43.37% weight loss occurred between 100–300 °C with a peak decomposition rate at a temperature of 251.35 °C, which could be assigned to the degradation of NHPI. In the second stage, 22.52% weight loss was observed between 300–400 °C, with a peak decomposition rate at 310 °C, which could be attributed to the decomposition of [bmim]­[OcOSO_3_]. On the other hand, the recycled SCILL-SILP system (Figure 13b) displayed a lower total weight loss of 26.97%, demonstrating partial retention of the mixture of IL and NHPI. Three major DTG peaks were observed at 68.84 °C, 195.92 °C, and 305.30 °C, corresponding to the distinct thermal decomposition stages. The first peak at 68.84 °C, with a minor rate of weight loss, could be attributed to the desorption of volatile compounds or moisture. The subsequent peaks at 195.92 °C and 305.30 °C indicate the thermal degradation of the retained NHPI and IL of the SCILL-SILP system. The ATR-FTIR spectrum of the recycled SCILL-SILP system (Figure 4d) further supports the TGA and DTG analyses, as the associated characteristic absorption bands were observed with reduced intensities.

**Figure 13:**
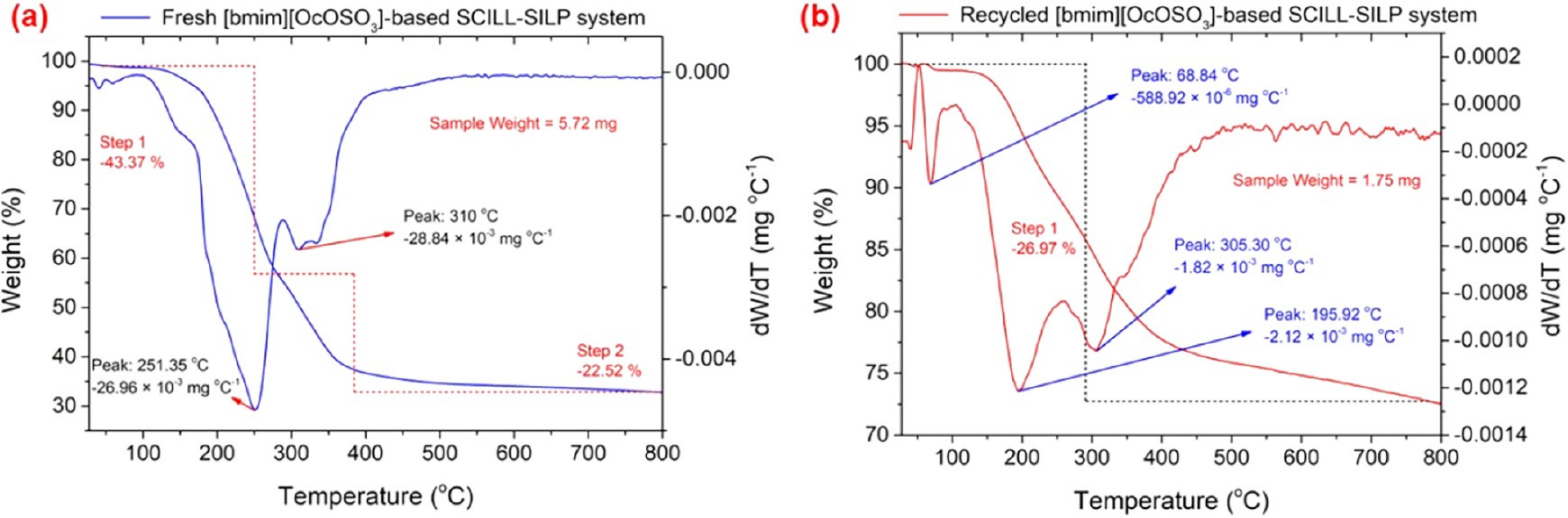
TGA and DTG curves, obtained for both the (a) fresh and (b) recycled SCILL-SILP catalytic systems after the second cycle.

### 3.5 Plausible Reaction Mechanism

A plausible reaction mechanism was proposed for the SILP (in the absence of Cu­(II)) and SCILL-SILP hybrid catalytic systems, which catalyzed the oxidation reactions of EB in a solvent-free environment using molecular oxygen as an oxidant (Figure 14). AIBN was utilized to produce the PINO radical from NHPI, which initiated the reaction. The generated PINO radical abstracts a hydrogen atom from the EB, yielding the 1-phenylethyl radical (PE) while regenerating the NHPI. In the following step, the produced PE radical quickly reacted with the absorbed oxygen to produce the 1-phenylethyl peroxyl (PEPO) radical, and the produced PEPO radical abstracted the hydrogen atom from the NHPI, regenerating the PINO radical while producing PEHP, and the cycle continued.^64^

**Figure 14:**
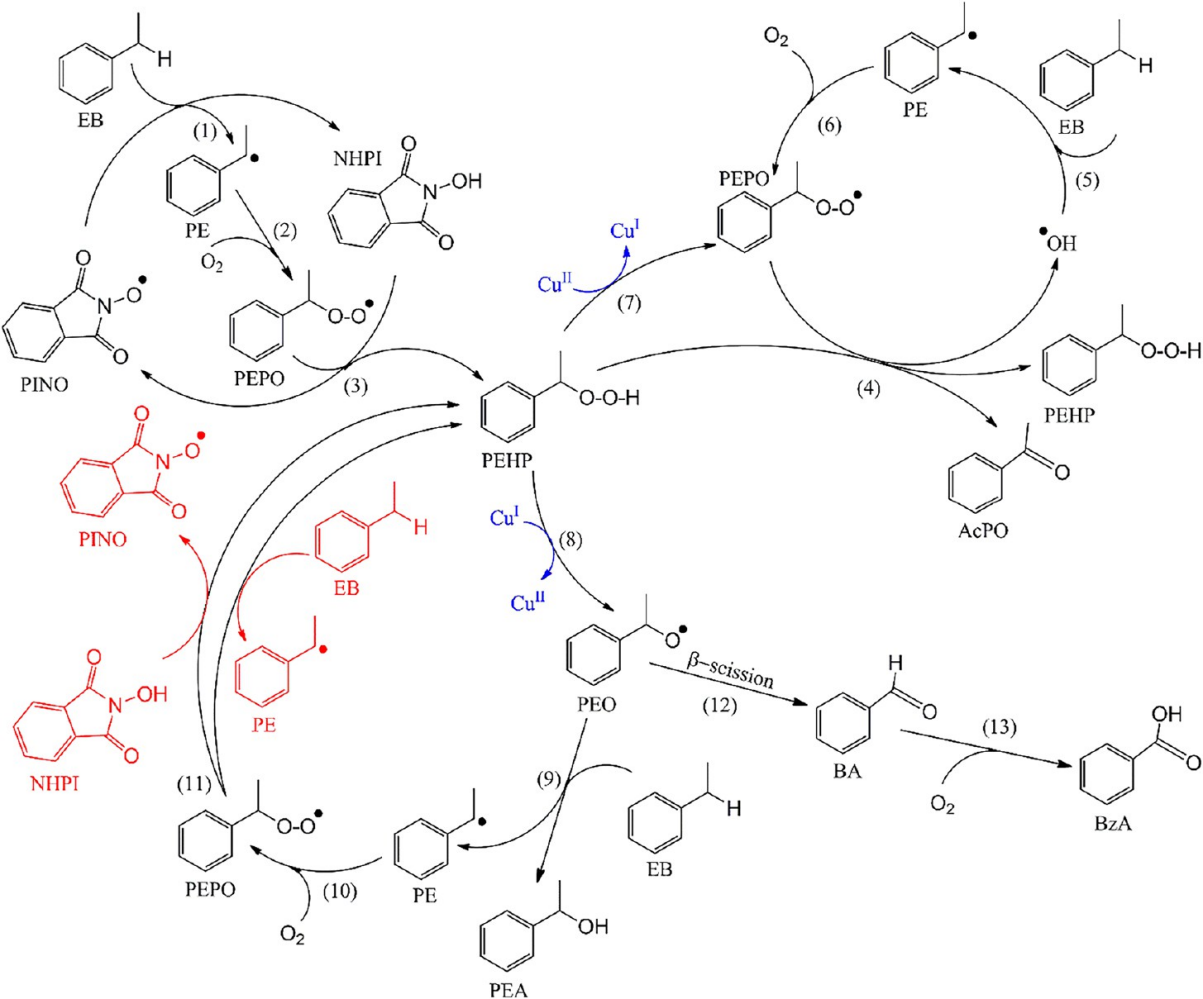
Plausible reaction mechanism of EB oxidation in the presence of the SILP (without Cu­(II)) and SCILL-SILP hybrid catalytic systems using molecular oxygen as an oxidant.

The produced PEHP is a crucial intermediate in the EB oxidation reaction and serves as the major chain propagator. PEPO can abstract the α-H atom of PEHP, and the PEHP promptly converts to AcPO and ^•^OH radical^25^ while reproducing PEHP. The PEHP can also decompose to PEO, which can react with EB, producing the PEA and the PE radical. The PE radical can react with oxygen, producing PEPO, and the produced PEPO can either react with EB or the PINO radical to produce PEHP again, and the cycle continues. The PEO can also undergo a β-scission reaction to produce BA, and the produced BA can further be oxidized, producing BzA. According to the results and discussion section, the amounts of BA and BzA produced are quite low, except for the [emim]­[OcOSO_3_] IL-based SILP and SCILL-SILP systems.

In PEHP decomposition, both the rate of reaction and product composition are influenced by the type of catalyst employed, i.e., the type of IL and MWCNTs, or MWCNT-COO-Cu. The high ketone to alcohol ratio obtained when MWCNT-COO-Cu was used indicates that the MWCNT-COO-Cu played a vital role in accelerating the decomposition of the PEHP into AcPO. It is consistent with the literature that among transition metal catalysts, Cu­(I/II) compounds accelerated the hydroperoxide decomposition and favored ketone formation.^5,89–92^ The comparison of various SILP and SCILL-SILP systems indicated that the presence of IL also favored the hydroperoxide decomposition to AcPO. It could be an effect of the higher polarity of the studied systems, which promotes the β-scission of alkoxy radicals more than the hydrogen atom abstraction reaction. Additionally, the solvent cage effect also affects the product composition.

### 3.6 Comparison with Literature Data

The results in this work have been thoroughly compared with literature data on the liquid-phase aerobic oxidation of EB (Table 3). The catalytic activity of the [bmim]­[OcOSO_3_]-based SILP system remained stable up to the second cycle, demonstrating its potential to serve as a metal-free and solvent-free catalyst under mild conditions for the aerobic oxidation of hydrocarbons. Similarly, the [bmim]­[OcOSO_3_]-based SCILL-SILP system demonstrated enhanced stability compared to other SCILL-SILP systems. Although the catalytic activity was decreased in the corresponding cycle, it still had higher activity compared to the corresponding [bmim]­[OcOSO_3_]-based SILP system. Furthermore, the catalytic systems developed in this study, based on CNTs, NHPI, and ILs, employ cost-effective and environmentally benign components, thereby aligning with the principles of sustainability and green chemistry.

**Table 3:**
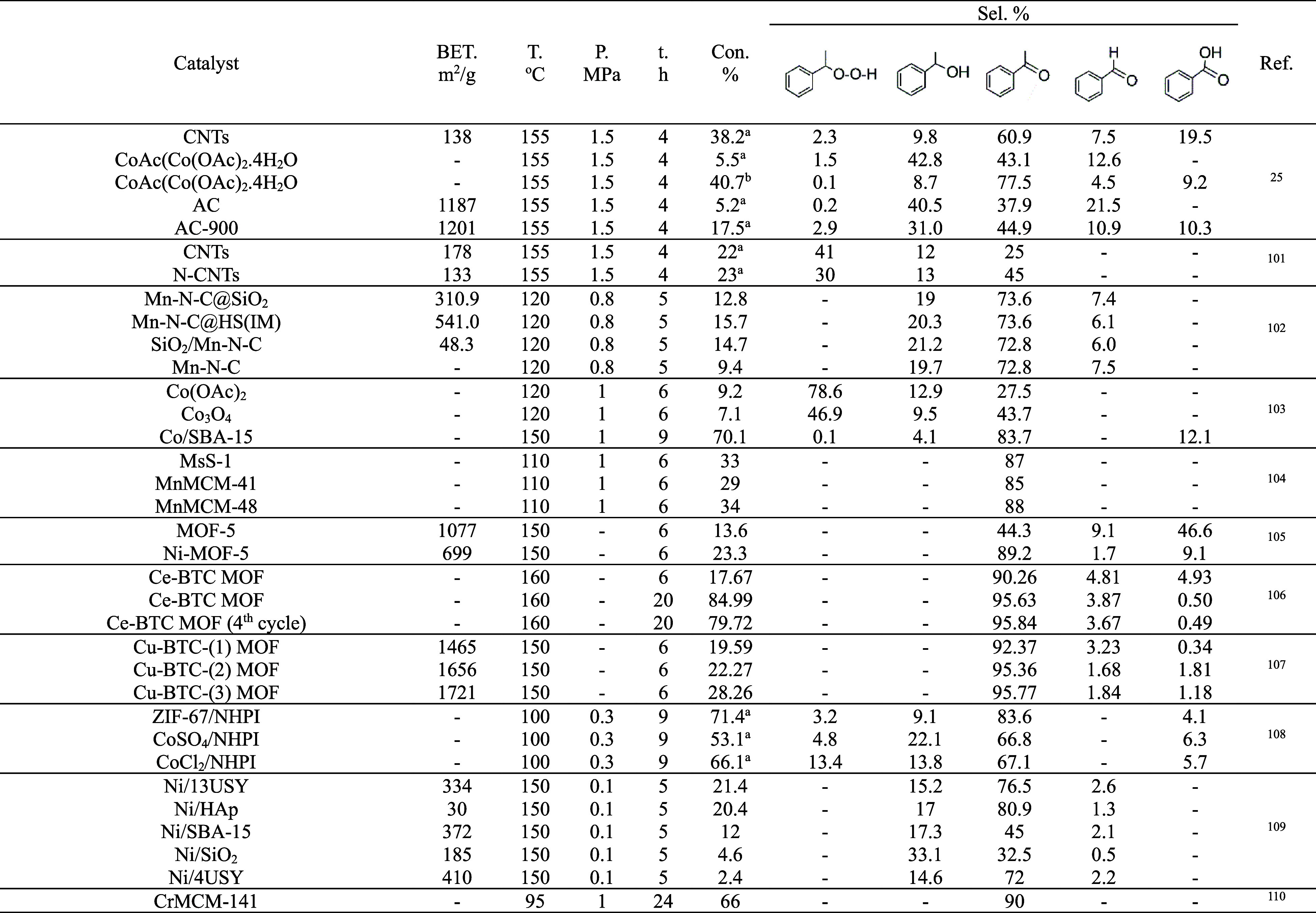

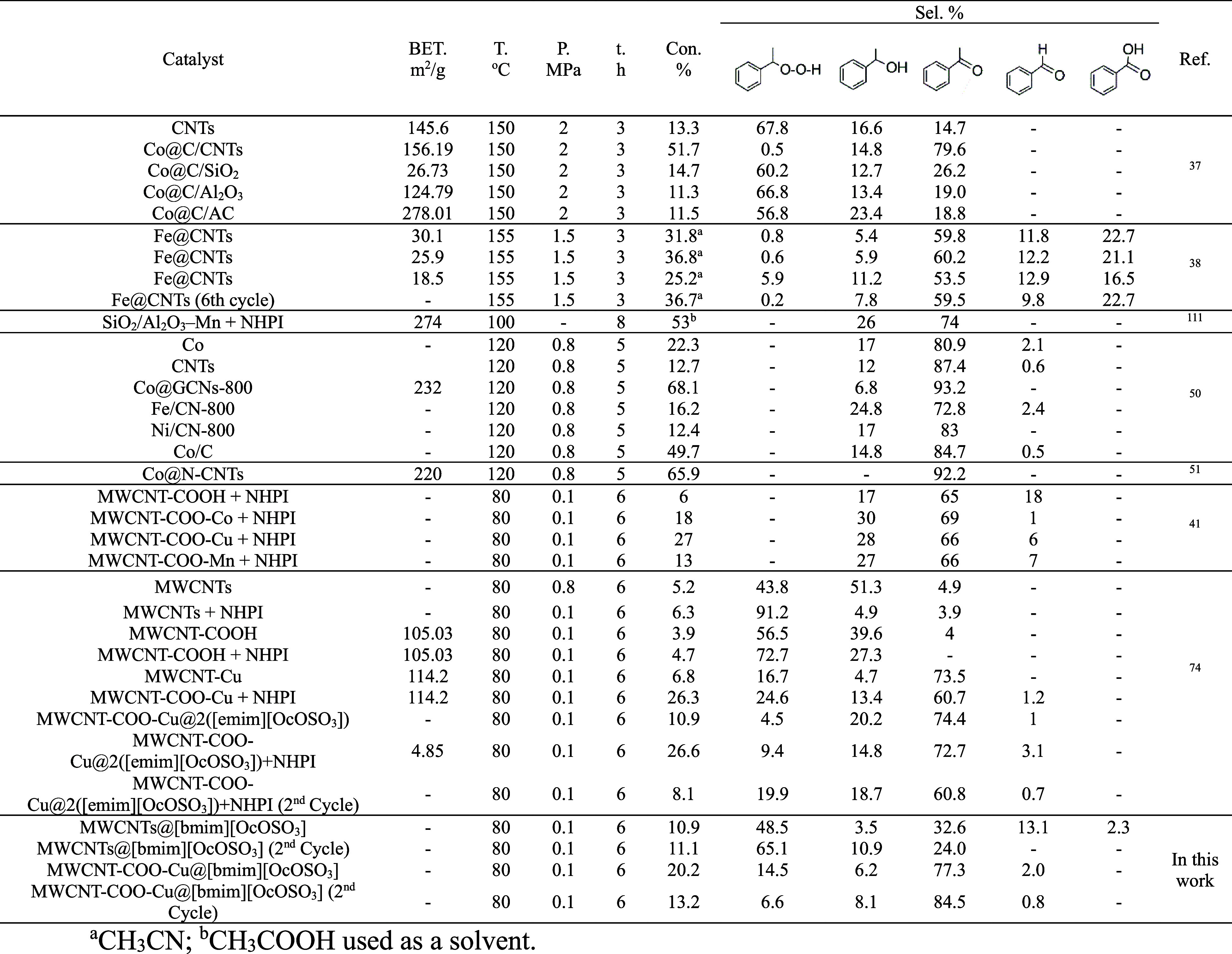
Comparison with Literature Data for the Liquid-Phase Aerobic Oxidation of EB^104–112^

a CH_3_CN

b CH_3_COOH used as a solvent.

## 4 Conclusion

In this study, pristine multiwalled carbon nanotubes (MWCNTs) and Cu­(II) functionalized multiwalled carbon nanotubes (MWCNT-COO-Cu) were employed as catalytic support for NHPI dissolved in various [bmim] cationic ILs to produce SILP and SCILL-SILP hybrid catalytic systems. The catalytic systems were employed in the solvent-free oxidation of EB using molecular oxygen.

It was demonstrated that the produced [bmim] cationic IL-based SILP systems were more active than pristine MWCNTs with and without NHPI. This could be due to the synergistic effect of the coating of the mixture of NHPI and ILs over the MWCNTs. The [bmim]­[Cl] IL-based SILP system had the highest catalytic activity, with an EB conversion of 12.2 ± 3.1% and a higher AcPO selectivity (84.1 ± 11.4%) compared with the other SILP systems. This enhanced catalytic activity was attributed to the halide anion in the [bmim]­[Cl] IL-based systems, which had a specific interaction with NHPI that accelerated the production of PINO radicals and decomposed the hydroperoxides at higher rates. An increase in the lipophilicity and/or chain length of the alkyl group in the [Cl] anionic-based ILs in the SILP systems increased the catalytic activity. The recyclability data showed that an increase in the lipophilicity of the IL in the SILP system drastically decreased the recyclability. However, the [bmim] [OcOSO_3_]-based SILP system was recyclable without significant loss of catalytic activity. The leaching test, TGA and DTG curves, and ATR-FTIR analyses of the fresh and recyclable [bmim]­[OcOSO_3_]-based SILP system showed that although the catalytic activity remained approximately the same, a portion of the mixture of IL and NHPI was leached in each of the corresponding cycles.

SCILL-SILP systems have lower catalytic activities compared to MWCNT-COO-Cu, which could be due to the higher viscosities of the coated ILs with dissolved NHPI, which covered the Cu­(II) active sites of the catalysts and prolonged the time of absorption and desorption of the reagents and products. Nonetheless, [bmim]­[NTf_2_] had the highest conversion of EB (22.6 ± 1.2%) compared with other [bmim] cationic IL-based SCILL-SILP systems. The recyclability data showed that the catalytic activities of all the SCILL-SILP systems decreased dramatically, but the percentage reduction in the catalytic activity for the subsequent cycle of the [bmim]­[OcOSO_3_]-based SCILL-SILP system was lower than that of the other systems. The leaching test, TGA and DTG curves, and ATR-FTIR analyses for the fresh and recycled [bmim]­[OcOSO_3_]-based SCILL-SILP system showed that a portion of the mixture of IL and NHPI was leached in the subsequent cycles. Furthermore, the SCILL-SILP catalytic systems have superior selectivities toward AcPO, which could be attributed to the solvent cage effect.

The decline in catalytic activities of the SILP and SCILL-SILP systems in the subsequent cycles could be associated with the leaching of the mixture of ILs and NHPI, as the leaching test, TGA and DTG curves, and ATR-FTIR analyses demonstrated that the systems were not completely heterogeneous, which could be due to the polarity of the reaction system. As the reaction time proceeded, more polar products formed and caused the leaching of the portion of the mixture of IL and NHPI. Future studies could be performed on the covalent immobilization of NHPI and IL on the surface of CNTs, which could prevent leaching.

## Data Availability

The data underlying this study are available in the published article.
